# The estimated annual financial impact of gene therapy in the United States

**DOI:** 10.1038/s41434-023-00419-9

**Published:** 2023-11-08

**Authors:** Chi Heem Wong, Dexin Li, Nina Wang, Jonathan Gruber, Andrew W. Lo, Rena M. Conti

**Affiliations:** 1 Research Affiliate, MIT Computer Science and Artificial Intelligence Laboratory & Department of Electrical Engineering and Computer Science, Cambridge, MA 02139 USA; 2 MIT Sloan School of Management and Laboratory for Financial Engineering, Cambridge, MA 02142 USA; 3 Graduate Student, Department of Economics, Princeton University, Princeton, NJ 08544 USA; 4 Undergraduate Student, Laboratory for Financial Engineering, MIT, Cambridge, MA 02142 USA; 5 Ford Professor of Economics, Chair, Department of Economics, MIT, Cambridge, MA 02142 USA; 6 Charles E. and Susan T. Harris Professor, Department of Finance, MIT Sloan School of Management, Director, Laboratory for Financial Engineering & CSAIL, MIT, Cambridge, MA 02142 USA; 7 Associate Professor and Dean’s Scholar, Department of Markets, Public Policy and Law, Questrom School of Business, and Co-Director Technology Policy and Research Institute, Boston University, Boston, MA 02115 USA

**Keywords:** Targeted gene repair, Drug discovery

## Abstract

Gene therapy is a new class of medical treatment that alters part of a patient’s genome through the replacement, deletion, or insertion of genetic material. While still in its infancy, gene therapy has demonstrated immense potential to treat and even cure previously intractable diseases. Nevertheless, existing gene therapy prices are high, raising concerns about its affordability for U.S. payers and its availability to patients. We assess the potential financial impact of novel gene therapies by developing and implementing an original simulation model which entails the following steps: identifying the 109 late-stage gene therapy clinical trials underway before January 2020, estimating the prevalence and incidence of their corresponding diseases, applying a model of the increase in quality-adjusted life years for each therapy, and simulating the launch prices and expected spending of all available gene therapies annually. The results of our simulation suggest that annual spending on gene therapies will be approximately $20.4 billion, under conservative assumptions. We decompose the estimated spending by treated age group as a proxy for insurance type, finding that approximately one-half of annual spending will on the use of gene therapies to treat non-Medicare-insured adults and children. We conduct multiple sensitivity analyses regarding our assumptions and model parameters. We conclude by considering the tradeoffs of different payment methods and policies that intend to ensure patient access to the expected benefits of gene therapy.

## Introduction

Gene therapy is a new class of medical treatment that alters part of a patient’s genome through the replacement, deletion, or insertion of genetic material to treat a disease. According to the United States Food and Drug Administration (FDA), there were four gene therapies approved for sale in the United States (U.S.) as of December 2022: voretigene neparvovec (marketed as Luxterna^®^) approved in 2017, onasemnogene abeparvovec-xioi (marketed as Zolgensma^®^) approved in 2019, brexucabtagene autoleucel (marketed as Tecartus^®^) approved in 2020, and etranacogene dezaparvovec (marketed as Hemgenix^®^) approved in 2022. While still in its infancy, gene therapy has the potential to treat and even to cure previously intractable diseases. For example, the introduction of voretigene neparvovec for inherited retinal disease and onasemnogene abeparvovec-xioi for spinal muscular atrophy (SMA) has already improved the lives of patients [1, 2]. Approval decisions are expected for gene therapies to treat sickle cell anemia, hemophilia A and B, certain types of leukemia, and other diseases in 2023.

The high price per treatment of available gene therapies as currently set by drug companies has raised concerns among payers, patients, physicians and policymakers. For example, at launch, voretigene neparvovec was priced at $425 thousand per eye, and onasemnogene abeparvovec-xioi was priced at $2.1 million per patient. From the perspective of the drug companies, charging high prices for their gene therapy is justified by their potential clinical benefit and the costs, risks and uncertainties of development. The clinical benefits include providing significant gains in longevity or symptom relief over conventional treatments and addressing often unmet needs in the treatment of rare diseases. In general, the clinical development of new drugs is time-consuming and costly. The process from the conception of a new drug to its clinical application may span decades and cost billions of dollars, with the bulk of the cost and time spent conducting later-stage clinical trials, even when these trials require fewer patients to complete than conventional small-molecule drugs [3, 4]. The process is also very risky, with only 13.8% of the therapeutic development programs which enter phase 1 of the approval process completing phases 2 and 3 and reaching approval by the FDA [5]. Sales and the use of new drugs also require the company to meet significant regulatory requirements for safety, efficacy and current good manufacturing standards. Most payers will only include new products under their coverage and reimbursement policies after receiving FDA approval.

New gene therapies, like many speciality drugs, are expected to have prices set by drug companies at levels that are too expensive for most patients to afford on their own [6]. Insurance coverage for gene therapies is also expected to vary. Many health plans do not cover the approved gene therapies that have already been launched into the commercial market, or they impose restrictive policies to limit the number of patients who might be treated with a given therapy in a year [7, 8]. If a patient who might benefit from treatment with gene therapy is uninsured [9] or underinsured [10] – i.e., when a person is covered by a health plan but faces substantial out-of-pocket costs in the form of deductibles and coinsurance payments – gene therapy is likely financially out of reach for them.

While the prices of individual gene therapies may be justified by their clinical benefits, spending in aggregate across all available gene therapies may be significant for payers, depending on the number of patients treated per year. Contemporaneous and systematic counts of the number of patients treated per year with existing gene therapies outside of clinical trials are not available; the drug companies marketing currently available gene therapies do not disclose this information in their shareholder reports. We are aware of only one academic study that has reported relevant estimates. Quinn et al. (2019) [11] estimate that the number of expected U.S.-based patients treated with gene therapy or stem cell therapy will amount to approximately 12 thousand in 2020 and over 340 thousand by 2030. The authors do not present their estimates disaggregated by gene therapy alone, nor do they provide estimates of expected patient counts by disease. Similarly, contemporaneous and systematic accounts of U.S. spending on available gene therapies by gene therapy, disease, or payer are not available. Insurers, such as Medicare, the taxpayer-supported health insurance for Americans over the age of 65, have not reported these statistics. While we do expect Medicare would pay for guideline-consistent use of gene therapy after FDA approval, we are less sure about payment by other health plans. Many non-Medicare health plans, especially those facing fixed annual budgets, may not be able or willing to absorb additional spending should a greater number of people become eligible for expensive gene therapies, or should many new expensive gene therapies reach the market [12]. Moreover, coverage and spending may lag in private health plans because a significant spending increase might have an impact on employee wages [13]. We are unaware of any academic study that has projected expected spending on gene therapies by U.S. payers.

This paper estimates the expected annual fiscal impact of gene therapy on the U.S. market. We do so by creating and implementing a novel financial model that estimates the future number of gene therapy approvals across all therapeutic classes, the size of their targeted patient populations, and their prices. We use advanced simulation methods to conduct the analysis. The use of simulation, rather than purely deterministic methods, allows us to capture the inherent risks and uncertainty in costs, revenues, and other parameters of this new therapeutic class. To populate the model, we include gene therapies already approved and marketed for sale in the U.S. or in late stage U.S. registered clinical trials underway as of December 2019. To assess the latter, we surveyed U.S. public clinical trial databases for late-stage gene therapy trials that were either actively recruiting or had completed patient enrollment as of December 2019. We use this information to estimate the expected annual number of gene therapy patients and their annual spending starting in January 2020 through 2034. Our estimates of fiscal impact are necessarily conservative because we do not account for gene therapies that entered human clinical trials in January 2020 or thereafter. Our analysis does account for products that entered into human clinical trials before this date, and it provides the best estimate available after taking into account the fact that trial registration and the recording of endpoints achieved by the trials are lagged. This assumption is similar to the modeling approach taken by Quinn et al. (2019). An added advantage of starting our projections in January 2020 is that it allows us to use sensitivity analysis to check the accuracy of our predictions based on our observations of market entry dates, expected patients treated, benefits from treatment, and product prices after January 2020.

## Materials and methods

### Human subjects and code availability

Please note, that this study is exempt from IRB review as it uses public and de-identified aggregate data. The code written to generate all estimates is available at the request of the study authors.

### Summary of methods

**Fig. 1:**
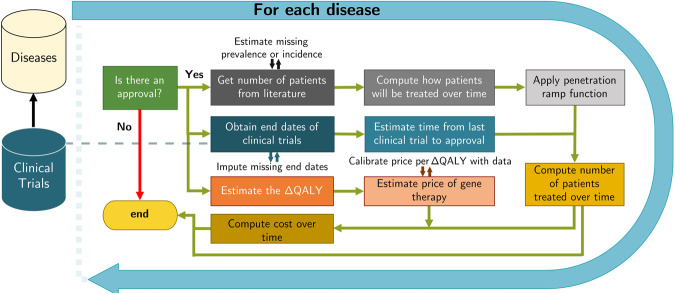
A flowchart showing the performance of the simulation. After extracting the information on each disease from the clinical trial databases, we simulate whether the disease will obtain an approval. If it fails to do so, the simulation will end for this disease in this iteration. Otherwise, we estimate the expected number of patients to be treated, compute the corresponding cost of treatment, and store the results. At each step of the computation, we sourced data from the published literature and impute missing information.

As outlined in Fig. 1, we first identify all existing late-stage clinical trials of gene therapies in phase 2/3 or 3 trials. We then estimate each trial’s likelihood of success, year of approval and spending on the successful therapies by summing the product of their expected prices and number of patients. We describe the separate tasks required for our analyses in the following subsections: [1] identification of the number of gene therapies currently in the clinical trial process and their associated diseases and therapeutic areas [2]; estimation of the probabilities of success of these trials [6]; estimation of the time to approval [7]; simulation of the expected number of patients treated by these therapies if approved; and [8] estimation of the expected market prices of the approved therapies.

#### Gene therapies in clinical trials and associated diseases and therapeutic areas

We use clinical trial metadata from the Citeline Trial Trove database and the U.S. National Library of Medicine’s ClinicalTrials.gov database to determine the number of gene therapies currently under development. We downloaded data from the Citeline database and isolated any trials tagged with ‘gene therapy’ under the ‘therapeutic class’ field. We supplemented this information by searching for trials on the *clinicaltrials.gov* main page using the keywords ‘gene therapy’, and then reading the trial description to determine if the trial was related to a gene therapy. All database queries were made on or before December 31, 2019. Clinical trials from both sources were merged before filtering for those clinical trials that were in either phase 2/3 or phase 3 of the development process and were not known to be ‘compassionate uses’ of the treatment. Compassionate use refers to the administration of investigational treatments outside of the clinical trial to treat patients with serious or immediately life-threatening diseases, or conditions when there are no comparable or satisfactory alternative treatment options. We exclude compassionate use from this study as those results are rarely used as data points in the clinical development process, and their uses often occur outside of clinical trial settings [14, 15]. Clinical trials without a U.S. trial site were included in the dataset because it is currently possible for the FDA to grant marketing approval using evidence from foreign clinical trials, as empowered by Federal administrative law 21 CFR Part 312.120 [16]. We removed repeated entries of the same trial. We then identified diseases, therapeutic areas, and patient ages targeted by each gene therapy.

This process yielded 109 unique trials investigating 57 distinct diseases, listed in Table A1 in the Supplementary Materials. We classified diseases into three categories: cancer (oncology), rare disease, and general disease. The distribution of diseases and the clinical trials by category and therapeutic area are shown in Table A1. Most trials and diseases were categorized in the area of oncology, followed by rare diseases. These therapeutic areas are notoriously risky for development. Only 3.1% of drug development programs in oncology and 6.2% in rare diseases go from phase 1 to approval, compared to the baseline of 13.8% across all drugs and indications [5].

#### Probability of success estimates simulation

We define a gene therapy development program as a set of clinical trials made by a sponsor when testing a therapeutic for efficacy against a disease. We considered whether gene therapy would be developed for a disease by simulating correlated random successes for each gene therapy program and observing if at least one approval took place. This computational method assumes that clinical trials are always perfectly correlated within the same development program. It can be argued that different gene therapy treatments for disease are highly correlated, since they operate on similar platforms (e.g., CAR-T or in-vivo gene delivery using adeno-associated virus vectors), even though different gene sequences may be targeted. To reflect this association, we assumed a correlation of 90% between development programs in our simulation. A sensitivity analysis, however, demonstrated that our computations are insensitive to this parameter.

Phase 3 to approval of probability of success (PoS_3*A*_) for each disease was informed by prior studies on the probabilities of success by therapeutic area of drug development programs from the MIT Laboratory of Financial Engineering’s Project ALPHA website [17]. These estimates were derived from over 55 thousand drug development programs between January 2000 and January 2020, and computed using the path-by-path method introduced in Wong et al. [5]. The PoS_3*A*_ values used in this study’s simulations are as follows: Autoimmune/Inflammation, 48.5%; Cardiovascular, 50.1%; Central Nervous System (CNS), 37.0%; Metabolic/Endocrinology, 45.7%; Oncology, 28.5% and Ophthalmology, 45.9%. The mapping of diseases to therapeutic areas is shown in Table A2.

#### Time to approval simulation

An estimate of the time to approval for gene therapy treatments was determined in order to assess the patient impact and cost over time of the treatment. Gene therapies require approval from the FDA through the biologics licensing application (BLA) pathway. Typically, companies submit a BLA to the FDA after the end of the clinical trial period. Our estimate assumed that the time between the end of the last clinical trial for the disease and the submission of the BLA was a variable drawn from a triangular distribution between 0 and 365 days, with a median of 182.5 days. This was informed by the practical knowledge that it takes an average of 6 months to prepare the documents for the BLA submission [5].

**Fig. 2:**
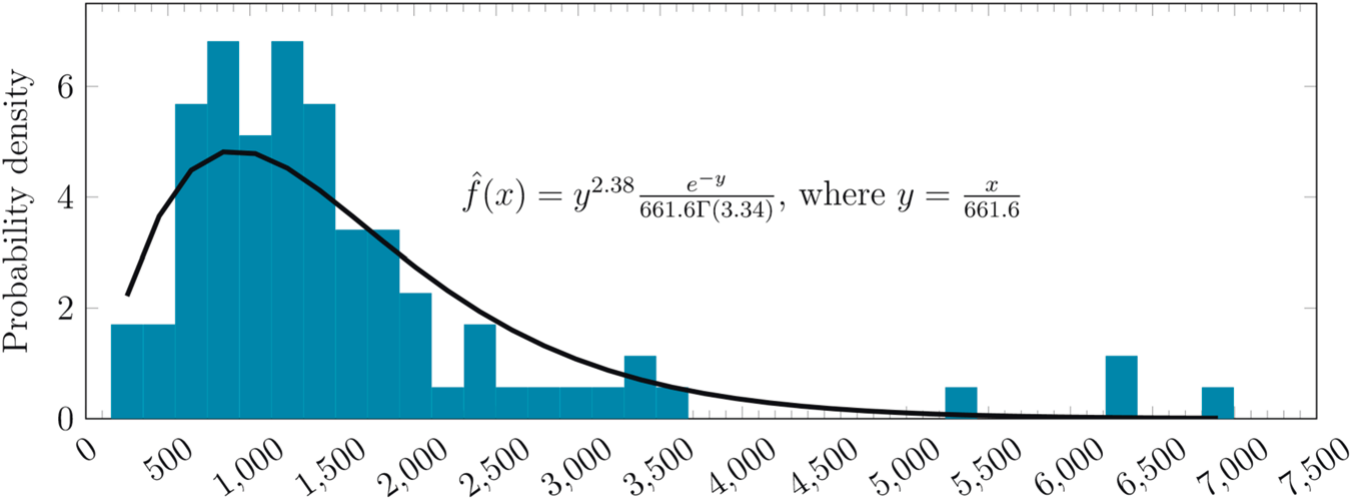
The empirical distribution of duration against our fitted gamma distribution.

There is an additional lag time between the submission of the BLA and the FDA’s decision. The FDA has 60 days to decide if it will follow up on a BLA filing [18], and it can take another 10 months to deliver its decision [19]. This implies the maximum possible time between BLA submission and FDA approval will be 12 months. Thus, our estimate assumed that the time between the BLA submission and the FDA decision would also be drawn from a triangular distribution between 0 and 365 days, with a median of 182.5 days. These assumptions are also valid for therapies that use the priority review pathways. This estimate also assumed that the BLA would be filed only after the last clinical trial for a disease had ended. Trials with missing declared end dates had their end dates imputed by adding random durations to the trial start date, drawn from a gamma distribution fitted to clinical trials with complete date information in the data (see Fig. 2).

Diseases with a prior approved therapy were automatically considered to be approved as of December 31st, 2020. For some diseases, their last clinical trial ended before January 2017, and no subsequent approval or product launch was observed. Diseases that matched this criterion were treated as though they had failed.

#### Number of patients simulation

This simulation captures the number of new and existing patients treated over time, conditioned on the disease receiving an approved gene therapy. We considered only the superset of patient segments listed in the clinical trials for each disease. For example, if there were two clinical trials, one targeting ‘patients above the age of 40’ and the other targeting ‘patients above the age of 18’, only the latter was considered when estimating the patient population for the disease. If insufficient information about the sub-population was given, it was assumed that all the patients with that disease were eligible.

#### Incidence and prevalence

We searched medical journals and online data repositories for the number of currently affected patients and the number of new patients per year for each indication, such as the Surveillance, Epidemiology, and End Results (SEER) website and *cancer.net*. If we identified an estimated patient population using this method directly, it entered our model; otherwise, we multiplied the prevalence and incidence rates of the disease by the population of the U.S., which was assumed to be 327.7 million [20].

In cases where estimates for the disease incidence were available but not the prevalence, we combined the incidence of the disease (i.e., *i* new patients a year) and the disease survival rate (i.e., *p*% of the people with a disease will be alive after *k* years) to obtain the steady-state estimate of the prevalence (*j*) using 1. Alternately, there were diseases identified where the prevalence was available but not the incidence. In these cases, we estimated the incidence from the prevalence by rearranging 1 to yield Eq. 2.

1 $${{{{{\rm{Prevalence}}}}}} \, ({{{{{\rm{j}}}}}})=\frac{{ki}}{1-p}$$

2 $${{{{{\rm{Incidence}}}}}} \, (i)=\frac{j(1-p)}{k}$$

To do so, we assumed that the number of patients would be constant through the years at a level *j* (that is*, ki* new patients are added over *k* years and *j*(1 − *p*) patients will die over the same period, and therefore *ki* = *j*(1 − *p*) will determine the number of patients that is constant over time). The number of patients for each disease is presented in Table A3. These estimates were adjusted to avoid double-counting in cases of overlapping patient populations, e.g., the number of patients for ‘Spinal Muscular Atrophy’ is the difference between ‘Spinal Muscular Atrophy’ and ‘Spinal Muscular Atrophy I’ (a sub-category of the former).

#### Treatment of patients over time simulation

This simulation assumes that newly diagnosed patients were treated immediately upon diagnosis and that the proportion of existing patients who seek treatment do so in such a way that the existing stock of patients will decline exponentially, with a half-life of *λ*. Mathematically, the proportion of existing patients that seek treatment between time *t* and *t* + *δ* after approval is given by *E* (*t, δ, λ*), where:

3 $$E\left(t,\delta ,\lambda \right)={e}^{\frac{-t{{{{\mathrm{ln}}}}}2}{\lambda }}-{e}^{\frac{\left(t+\lambda \right){{{{\mathrm{ln}}}}}2}{\lambda }},t \, > \, 0$$

We assumed that 25% of the existing stock of patients would seek treatment in the first year of this simulation. This required that the half-life be set to 28.91 months, which in turn implied that 95% of all patients who were diagnosed prior to the approval of the gene therapy would want treatments within 10.5 years. A sensitivity analysis was performed on this assumption to determine its impact on the results.

#### Patient penetration simulation

It is unlikely that all patients with a prevalent case of a disease will receive gene therapy treatments. This may be due to ineligibility, or lack of awareness of the treatment, among other reasons. We labelled the percentage of the patients that received gene therapy treatments in any given period as the ‘patient penetration rate,’ and modelled this rate using a ramp function, *ρ* (*t*, Θ_*max*_*, T*_*max*_). The ramp function is frequently used by industry to model the rate of adoption of a product or technology [11]. It is given by:

4 $$\rho \left(t,{\Theta }_{\max },{T}_{\max }\right)=\left\{\begin{array}{c}\frac{{t\cdot \Theta }_{\max }}{{T}_{\max }},0\le t\le {T}_{\max }\\ {\Theta }_{\max },{{{{{\rm{otherwise}}}}}}\end{array}\right.$$

We assumed different ramp functions for diseases belonging to our three categories: rare disease, ‘general’ or chronic disease, and cancers. For rare diseases, faced with improved prospects of survival, we assumed more patients would be willing to enrol in new treatments quickly after approval. In addition, since the number of patients with individual rare diseases is relatively small, insurers may be more willing to cover these therapies and manufacturers more able to cope with a larger proportion of patients. Given this, *µ*_*θ*_ was assigned a high value of 40% and *µ*_*T*_ was assigned a low value of 6 months.

On the other hand, many chronic diseases in our general category are seldom deadly, while affecting a larger number of patients, even in the millions. Since an acceptable standard of care is often available for these conditions, patients may be less inclined to use new treatments due to a lack of certainty in benefit and durability. Thus, this study assumed that the maximum penetration rate for gene therapies approved to treat general chronic diseases would be 1%, and the ramp-up period, 5 years.

As an intermediate case, cancers have characteristics that fall between these two extremes, but in general, they are more like the rare disease category. We therefore assigned values of 10% to the maximum penetration rate and 12 months to the ramp-up period. All variances were set to 10% of their means in order to model a moderate level of uncertainty in our numbers. This assumption did not affect our mean estimates of the number of affected patients or spending on gene therapy.

The net number of patients to be treated for the disease at time *t* after the approval of a gene therapy is given by:

5 $${{{{{{\rm{Patients}}}}}}}_{t}=\rho \, (t,{\theta }_{\max },{T}_{\max }){{\cdot }}[{{{{{\rm{New}}}}}} \, {{{{{{\rm{patients}}}}}}}_{t}+E (t,\delta ,\lambda ) {{\cdot Existing}} \, {{{{{{\rm{patients}}}}}}}_{t}]$$

We did not consider the effect of market competition among different therapies for the same disease, or the effect of patient type on the expected number of treated patients. This is in part because it is hard to determine the expected patterns of use without an existing approval. Instead, we modelled each treatment-disease fraction of the population that would be eligible for treatment, assuming independence.

#### Expected market pricing simulation and QALYs gained from gene therapy treatment

The cost to the healthcare system of providing the gene therapy for a disease for all patients treated at time *t* after approval is given by *C*(*t*), where

6 $$C\left(t\right)={{Patients}}_{t}\times {{{{{\rm{Priceof}}}}}}\; {{{{{\rm{gene}}}}}}\; {{{{{\rm{therapy}}}}}}$$

The price of each treatment is crucial to computing the expected total spending, and a source of considerable uncertainty because the gene therapies that are the subject of this analysis are generally not yet approved, and consequently not priced by their company. We address this uncertainty by estimating expected prices using well-established methods. The Institute for Clinical and Economic Review (ICER) is an independent nonprofit organization that evaluates the clinical and economic value of healthcare innovation. ICER calculates the expected prices of new therapies based on the relative benefits and costs to the patient reported in pivotal clinical trials. ICER does this by comparing the expected quality-adjusted life-year (QALY) with and without the treatment, then multiplying the difference in QALY (∆QALY) by a constant value of a life-year gained, typically set between $50 thousand and $150 thousand per ∆QALY [21].

7 $${{{{{\rm{Price}}}}}}{{{{{\rm{of}}}}}}\; {{{{{\rm{gene}}}}}}\; {{{{{\rm{therapy}}}}}}={{{{{\rm{Price}}}}}}\; {{{{{\rm{per}}}}}} \, {{{{{\rm{QALY}}}}}}\times \Delta {{{{{\rm{QALYs}}}}}}$$

At the time of our analysis, ICER had published several reports containing estimates of QALYs gained by patients treated with existing gene therapies, including those with vision loss associated with biallelic RPE65-mediated retinal disease following treatment with voretigene neparvovec^®^ [2], and with SMA Type I following treatment with onasemnogene abeparvovec-xioi^®^ [22]. These reports computed the ∆QALY using the results of the clinical trials that formed the basis for FDA approval to estimate the potential improvements in the quality of life and life expectancy of the patients among treated patients. Replicating the ICER method for all the clinical trials under consideration in this paper was infeasible since most of these trials were not yet complete nor had they reported pivotal trial results for FDA approval during the timeframe of our analysis. As an alternative, we developed a mathematical model based on a modification of the ICER method and calibrated using the pricing of currently available gene therapies approved for use in the U.S. market, to estimate the expected increase in QALYs from gene therapy for each disease in our sample.

The Appendix describes our method for estimating the expected QALYs gained from gene therapy. The next subsection describes our method of estimating the price per QALY gained from gene therapy.

#### Price per ∆QALY

To estimate as realistic a market price of new gene therapy as possible, we calibrated our assumed price per ∆QALY with the two currently available gene therapies priced in the U.S. market as of January 2020: onasemnogene abeparvovec-xioi, priced at $2.1 million per patient [23], voretigene neparvovec, priced at $0.425 million per eye treated [24], Separately, betibeglogene autotemcel, marketed as Zynteglo, and sold at a cost of 1.6 million Euros (approximately $1.8 million), has been approved in the European Union at the time of our analysis, and was approved in the U.S. in August 2022 with a price of $2.8 million for a one-time dose. To improve the precision of our estimates, we added the two CAR-T therapies also approved and available in the U.S. market, tisagenlecleucel, marketed as Kymriah and approved in 2017, priced at $0.475 million for a one-time dose [25], and axicabtagene ciloleucel, marketed at Yescarta and approved in 2019, priced at $0.373 million for a one-time dose [25]. We calibrated the price per ∆QALY by minimizing the mean-squared error (MSE) between the estimated price given the expected change in QALY and the actual price. We reported the mean absolute percentage error (MAPE) between the estimated price and the actual price in addition to the MSE. To account for potential ∆QALY differences between the gene therapies and the CAR-T therapies, we performed two separate calibrations. We assumed that the price per ∆QALY for general diseases was identical to that for cancerous indications.

Considering only the therapies approved in the U.S. through January 2020, we estimated a price per E(∆QALY) of $101,663 (MSE: 2.18 × 10^9^, MAPE: 11.2%) for rare diseases and $40,797 (MSE: 1.77 × 10^10^, MAPE: 44.2%) for other diseases. Using all the data points, the price per E(∆QALY) for rare diseases increases to $114,781 (MSE: 1.70 × 10^12^, MAPE: 108%). In this paper, we used the former value in our calculations, since it has a smaller MSE and better reflects current prices in the U.S. This value gives us pricing estimates of $2.09 M per patient for onasemnogene abeparvovec-xioi and $0.470 M per eye for voretigene neparvovec, which is consistent with the prices we observe in the real world.

Our calibrated price per E(∆QALY) for cancerous indications is just slightly below ICER’s $50 thousand to $100 thousand range for ‘intermediate care value’. The higher price per E(∆QALY) for rare diseases reaffirms the general belief that developers of treatments for rare diseases should be compensated more for their elevated research and development risk and the lower financial prospects of serving a small population of patients. It is assumed that the clinical cost of delivering the gene therapy is a negligible fraction of the overall cost of development (though it is considerably higher than the delivery cost of conventional therapeutics). It is also likely that the outside option cost will be similar.

The expected increases in QALY computed by our model were also close to those reported by ICER for these treatments [1, 2]. For example, we estimated that treatments for Spinal Muscular Atrophy Type 1 and Leber Congenital Amaurosis due to RPE65 Mutations provided 20.56 and 4.63 incremental QALYs, whereas ICER estimates onasemnogene abeparvovec-xioi and voretigene neparvovec to provide 12.23 to 26.58 and 1.3 to 2.7 incremental QALYs, respectively.

ICER also provides a range of ∆QALY estimates corresponding to different age groups. To compare our estimates, we followed their definition of age groups: a minor is defined as a patient below the age of 18, and an elderly patient as one who is older than 62 years old. The remaining cohort of the patients was defined as adults. We used the distribution of ages to produce a weighted average estimate. We deliberately applied the same methods and assumptions for all other diseases to estimate the expected changes in QALY for Spinal Muscular Atrophy Type 1 and Leber Congenital Amaurosis due to RPE65 Mutations, even though these numbers were directly available from ICER reports. This calibration of price per ∆QALY corrects for potential biases in our data, and as a result, allows our price estimates to be more realistic.

Finally, one million iterations of our simulation were performed to compute the mean number of gene therapy patients and their total spending. At this number of iterations, the computed mean was expected to be within 1.89% of the true mean 95% of the time. The 5th and 95th percentiles of the computed values were reported as the upper and lower bounds respectively.

### Sensitivity analyses

To test the sensitivity of our results to initial conditions and assumptions, we simulated ±20% changes in the following variables and analyzed their impact on our results:

1. The maximum penetration rate in the ramp function, Θ_*max*_
2. The time to maximum penetration rate in the ramp function, *T*_*max*_
3. The amount of QALY gained in each disease
4. The price per ∆QALY
5. The phase-3-to-approval probability of success (PoS_3*A*_)
6. The number of new patients of each disease
7. The number of existing patients of each disease
8. The time from phase 3 to BLA
9. The time from BLA to approval

For each of these factors, we considered its impact on the peak monthly spending and the cumulative spending from January 2020 to December 2034 of patient treatment. We explored how the variables might change the timing of peak monthly spending. Additional details are provided in the Appendix.

## Results

### Expected number of approvals and patients

**Fig. 3:**
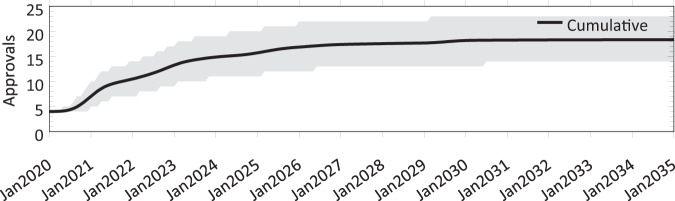
Cumulative number of approvals between January 2020 and December 2034 observed from 1,000,000 simulation runs. The line represents the mean and the shaded region represents the 5th and 95th percentiles of our simulation.

Based on the assumptions detailed in the previous section, the results of our simulation suggest that the expected number of gene therapies approved between January 2020 and January 2034 will be 18.3, with a 90% confidence interval of (14.0, 23.0) (see Fig. 3).

**Fig. 4:**
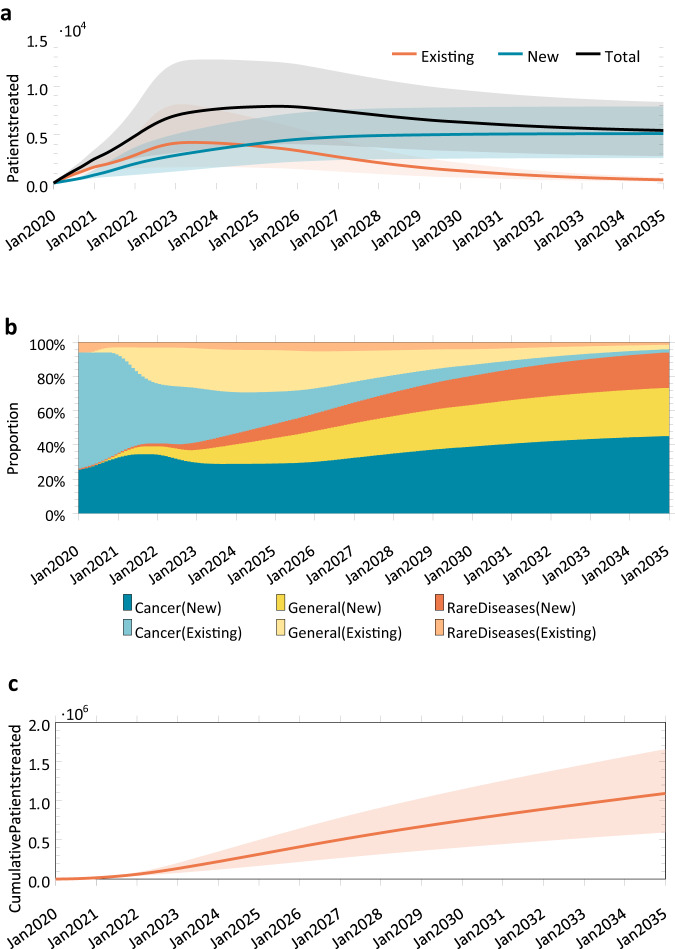
Number of patients treated between January 2020 and December 2034, obtained from 1,000,000 simulation runs. **a** Monthly number of patients treated with gene therapy across all diseases, among existing and new patients. **b** Stacked chart depicting the proportion of existing and new patients treated in that month, by disease category. **c** Cumulative number of patients treated. The line represents the mean and the shaded region represents the 5th and 95th percentiles of our simulation.

The number of patients treated by month is shown in Fig. 4a. These simulations expect the number of treated patients to peak around 7911 per month in July 2025 (CI: [3978, 12,477]) before declining to 5424 by December 2034 (CI: [2778, 8350]). The monthly number of existing patients treated exceeded the monthly number of newly diagnosed patients treated until September 2024, when this trend is expected to reverse. Only 7% of all patients treated in December 2034 were preexisting patients. Cancer patients were expected to form the largest group of patients receiving gene therapy treatments, simply due to the number of cancer indications being targeted (see Fig. 4b). The relative proportions of cancer, general disease, and rare disease patients are expected to be 48.0%, 30.0%, and 22.0%, respectively, in December 2034. The cumulative number of patients to be treated is expected to be 1.09 million (CI: [0.595 M, 1.66 M]) by the end of December 2034 (see Fig. 4c).

The results suggest the expected number of new patients treated with gene therapies will grow from 16,244 in 2020 to 94,696 in 2025 before declining to 65,612 in 2034. The decline can be attributed to the declining stock of existing patients as they are treated, and the fact that in our analyses new development programs that entered human clinical trials after December 2019 were not considered. We also estimate the annual number of patients over time by age group (not shown). The proportions of patients who were classified as minors, adults, or elderly were 17.9%, 35.4%, and 46.7% respectively.

### Expected spending

**Fig. 5:**
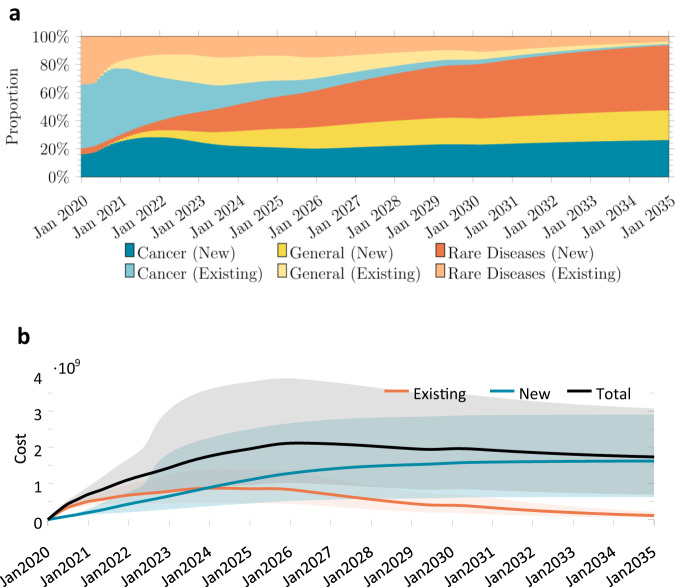
Simulated monthly spending on patients treated with gene therapy. **a** Monthly spending on treating existing and new patients with gene therapy. **b** Stacked chart depicting the proportion of spending on treating existing and new patients in that month, by disease category. The line represents the mean and the shaded region represents the 5th and 95th percentiles from our simulation.

We expect an increase in spending up to $2.11 billion per month (CI: [1.01B, 3.88B]) in April 2026, before decreasing slowly to a steady-state rate of $1.62 billion (CI: [0.624B, 2.9B]) per month (see Fig. 5a). This decline in part reflects the fact that our simulations analyze a fixed stock of gene therapies already approved or under development on or before December 2020. Treating existing cancer patients will initially consume over 45.6% of the total monthly expenditure, but will decline to only 0.99% by December 2034 (see Fig. 5b). In contrast, the proportion of spending on new patients in the ‘general disease’ and ‘rare disease’ groups will increase from 0.0% and 4.26%, respectively, in February 2020, to 21.2% and 46.2% by December 2034. The monthly spending on treating existing patients is projected to exceed the monthly spending on treating newly diagnosed patients by November 2023. The cumulative discounted spending on treating patients with approved gene therapy products is expected to reach $241 billion (CI: [123B, 402B]) by December 2034.

**Fig. 6:**
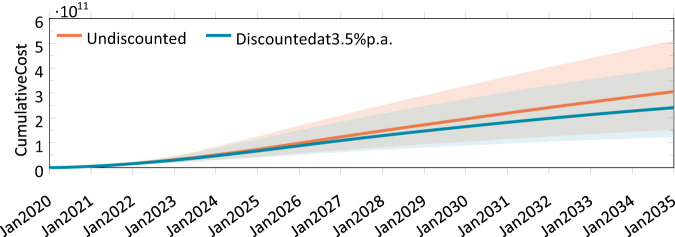
Cumulative spending on treating patients with gene therapy. The line represents the mean and the shaded region represents the 5th and 95th percentiles of our simulation.

In terms of annual spending on approved gene therapies, we estimate that approximately $5.15 billion would be spent in 2020, increasing to $25.3B in 2026, before declining to $21.0B in 2034 (see Fig. 6). Across all years in our models, average annual spending on gene therapies amounts to $20.4 billion.

**Fig. 7:**
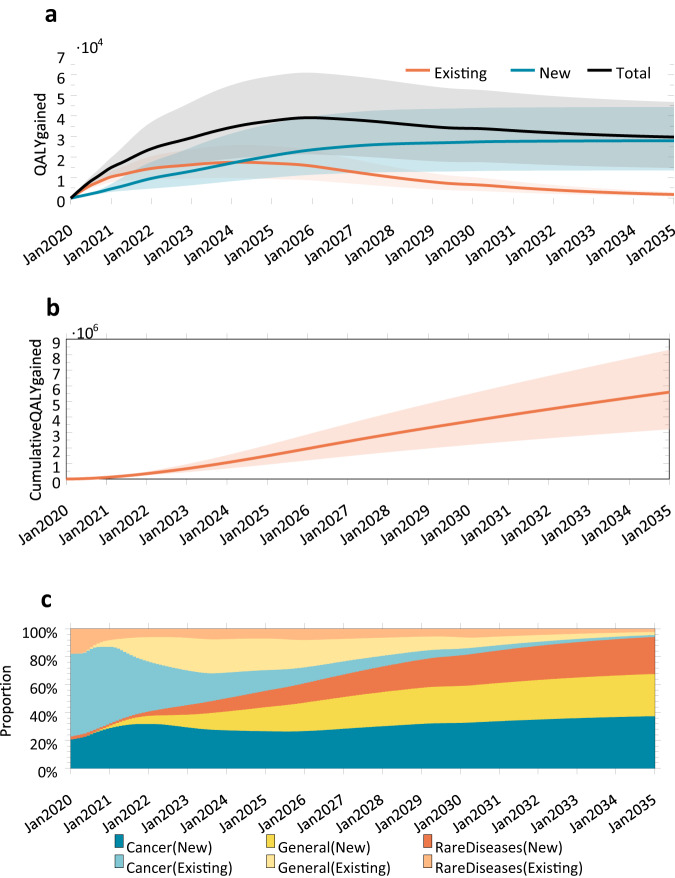
QALYs gained by treating existing and new patients with gene therapy. **a** QALYs gained by treating existing and new patients with gene therapy. **b** Cumulative QALY gained by treating patients with gene therapy overall. **c** Cumulative QALY gained by treating patients with gene therapy broken out by new and existing patients. The line represents the mean and the shaded region represents the 5th and 95th percentiles of our simulation.

The average expected increase in QALYs from gene therapy treatments is estimated to be 5.12 life-years per treated patient (see Fig. 7a–c). Using an average cost at 2020 present value, this gain amounts to $43,110 per unit change in QALY.

Minors, adults and the elderly will consume 43.2%, 26.0%, and 30.9%, respectively, of the annual average spending. If we apply these age group percentages to expected insurance coverage, we can estimate the expected proportion of spending on gene therapy by insurers in the U.S. For this analysis, we assume all elderly people will be covered by Medicare, while two in five children and one in seven adults will be the U.S. are covered by Medicaid. The remainder of insurance coverage will be from private sources related to employer-sponsored plans, exchange plans or individual plans [26]. Using these proportions, we estimate that the expected annual spending by Medicare, Medicaid and private sources respectively may reach $8.1, $5.44, and $12.2 billion (results not shown).

### Results of sensitivity analysis

**Fig. 8:**
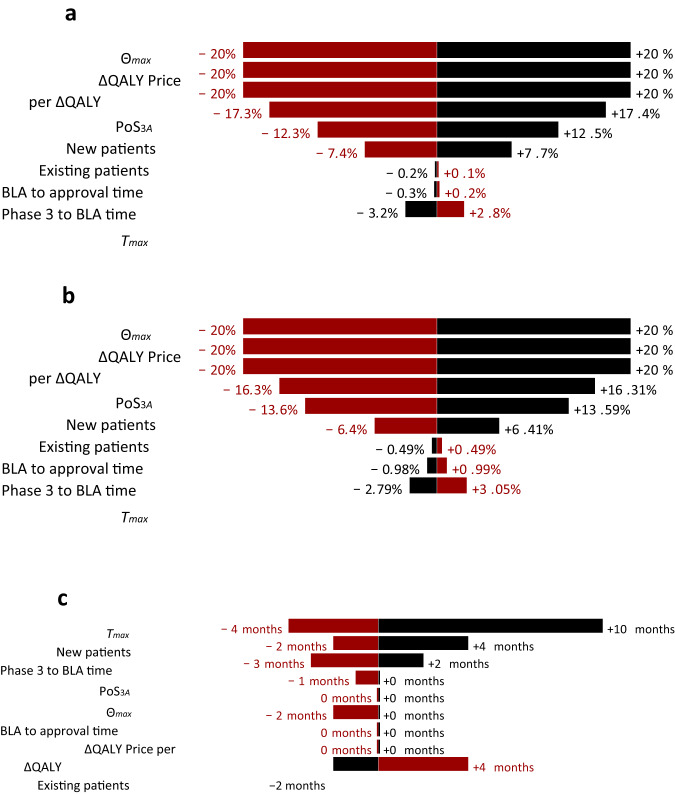
Tornado charts showing the sensitivity of the variables on the different metrics. **a** Tornado chart of the impact of the variables on the peak value. **b** Tornado chart of the impact of the variables on the cumulative spending (both nominal and discounted). **c** Tornado chart of the impact of the variables on the date of peak value. Since we compute by calendar month, a small machine precision error may change the results by 1 month. The black bars represent the effect of increasing the variable by 20% and the red bars represent the effect of decreasing the variable by 20%.

As can be seen from Fig. 8a–c, the percentage change in the discounted cumulative spending and the maximum monthly spending on treating all patients with gene therapy scale linearly with the percentage change in several variables: the maximum penetration rate (Θ_*max*_), the QALY gained (∆QALY), and the price per ∆QALY. Increasing or decreasing the transition probability from phase 3 to approval, or the number of new or existing patients leads to sublinear increases or decreases in the discounted cumulative spending and the maximum monthly spending. However, changing the time variables, such as the number of days from phase 3 to BLA, from BLA to approval, or the ramp-up period (*T*_*max*_), induces a small change in the opposite direction.

Introducing perturbations of 20% in the probability of success, the number of new patients, the number of days from Phase 3 to BLA or from BLA to approval, or the time to maximum penetration rate in the ramp function (*T*_*max*_) will change the date of the peak monthly spending in the same direction as the perturbation, by up to 10 months. Increasing or decreasing the number of existing patients, on the other hand, will cause a shift of up to 4 months in the date of peak spending in the opposite direction. Perturbing the maximum penetration rate (Θ_*max*_), the QALY gained (∆QALY), and the price per ∆QALY will not change the date of peak spending.

We also studied the effect of changing our assumption regarding the correlation between development programs. Changing the correlation from our assumed value of 0.9 to 0 (i.e., perfectly uncorrelated development programs) increases the mean discounted cumulative spending by 3.4%, from $241 billion to $245 billion. Increasing the correlation to 1.0 instead will decrease the mean discounted cumulative spending by 0.4% to $236 billion.

In addition, we varied the proportion of existing patients seeking treatment in the first year, which determined the *λ* parameter in Eq. 3, and observed that mean discounted cumulative spending changes by between −32% and +0.08%. We expect the results to differ by less than 5% from our baseline if the proportion of existing patients seeking treatments in the first year is between 8% and 45%.

As an additional check, we verified that the two additional gene therapies that were approved and launched in the U.S. market between January 2020 and June 2021 (brexucabtagene autoleucel for adult leukemia, July 2020, $.373 million for a one-time dose and etranacogene dezaparvovec for adult hemophilia B, March 2021, $.482 million for a one-time dose) were priced within ICER recommended ranges. This provides additional credence to our general method.

**Fig. 9:**
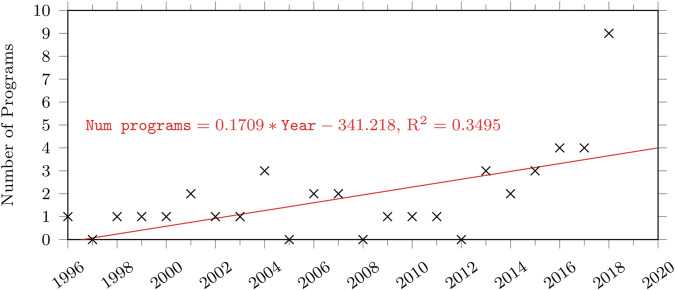
A plot of the number of programs initiated over time in our dataset.

We also simulated future gene therapy programs that might enter the pipeline and compared the results against our baseline (see Fig. 9). To do so, we fit a linear equation of the number of gene therapy programs against the year. We extrapolated the fitted line to obtain the expected number of new gene therapy programs entering the pipeline between 2020 and 2034.

We modelled the occurrence of new gene therapy programs with a Poisson process, with the time between occurrences following an exponential distribution. More precisely, if *κ* programs will be expected in a year, the time between consecutive events (*t* > 0) will be a probability density of $\kappa {e}^{-\kappa t}$. For every event, we randomly assign a disease classification before simulating the success of the program, and if successful, its time of approval, the number of patients and spending over time.

**Fig. 10:**
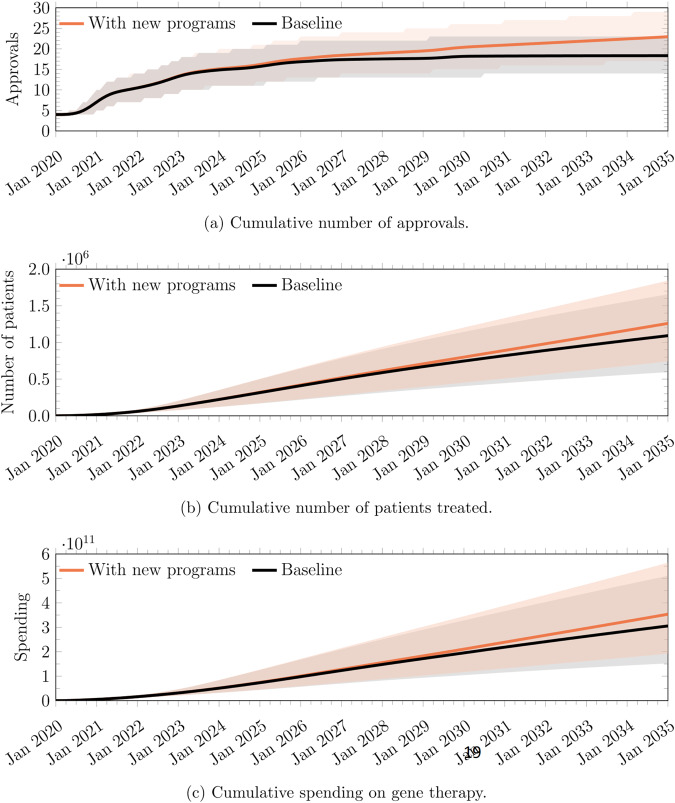
Comparison between the results of the simulations with and without assuming additional gene therapy programs entering the pipeline. **a** Cumulative number of approvals. **b** Cumulative number of patients treated. **c** Cumulative spending on gene therapy. The line represents the mean and the shaded region represents the 5th and 95th percentiles of our simulation.

As can be seen from Fig. 10, while introducing new gene therapies into the pipeline increased the cumulative number of approvals by 25.1% from 18.3 to 23.0, the cumulative number of patients increased by only 15.3%, from 1.09 M to 1.26 M. Similarly, our cumulative spending estimates only increased by 15.7%, from $306B to $354B.

## Discussion

We estimate that the annual spending on gene therapy, using the gene therapies already approved and launched in the U.S. market and the pipeline of gene therapies in late-stage clinical trial development as of December 2020, will average $20.4 billion. This estimate may be a lower bound since our simulation employs conservative assumptions about the speed and volume of gene therapy development. Specifically, we did not account for the possibility that a program in Phase 1 or Phase 2 might be fast-tracked or granted accelerated approval. Extrapolation of the number of new development programs increased our mean estimated spending and number of patients by approximately 16%, although the effects could be much larger in the long run.

A potential criticism of our approach is that estimating the prices of new gene therapies based solely on changes in QALY will both overestimate and underestimate aggregate spending. On the one hand, we did not consider the potential cost savings of gene therapy due to the lack of necessity of multiple therapeutic sessions over time compared to the current standard of care, nor did we consider the recovery of the opportunity cost of caregivers. While gene therapy may provide net cost-savings in treatment (e.g., valoctocogene roxaparvovec for the management of hemophilia A [27]), the evidence is not robust at this time. We also have not considered the possibility that multiple gene therapies for the same disease may lower their prices. Examining the clinical trial information available to us, we do not see overlapped gene therapy programs targeting the exact same disease-population pair, reducing the potential for very robust brand-brand competition. There is also no empirical evidence that the presence of multiple brand-name drugs in the same therapeutic class lowers the prices of existing drugs [28].

On the other hand, we have omitted the clinical costs of delivering gene therapy in our analysis, which are often higher than conventional therapeutics due to the need for inpatient hospital care. In addition, research has shown that new medical technologies generally raise health costs and that cost-increasing changes in treatments outweigh cost-saving changes the majority of the time [29, 30]. These considerations suggest that our approach is conservative and that our estimates are likely to be lower bounds for the realized annual spending over the time period of study.

Our estimates suggest the average cost of gene therapy to amount to $43,110 per unit QALY, several times the average annual expenditure of $16,346 for American cancer patients between 2010 and 2014 [31]. However, when viewed from the broader perspective of aggregate U.S. spending, these figures seem less daunting. In 2018, the U.S. tax revenue was $3.33 trillion, of which individual income tax and payroll tax revenues were $1.68 and $1.17 trillion, respectively [32]. Fully funding the average annual spending of $20.4 billion through income and payroll taxes will require an increase of 0.612%. Since Medicare already covers all elderly patients, we estimate that the program would need to increase its annual budget by up to $7.89 billion, or 1.1% of its 2018 spending of $750.2 billion [33]. Funding this increase would require either an increase in payroll taxes or a reduction in other expenditures.

We estimate that annual gene therapy spending by Medicaid may reach $5.44 billion. This is approximately 0.9% of its 2018 spending of $597.4 billion [33]. Since Medicaid cannot restrict access to therapies with expected benefit to the patient, while state budgets must be balanced year to year, managing an expected increase in spending on new treatments will require either raising funds from state and federal governments to pay for these additional costs or cutting benefits. We estimate that the annual spending by minors and adults insured by private health plans and employers on gene therapy may reach $12.2 billion. This amount of annual spending will also likely pose significant challenges for healthcare plans and employers to manage. In order to manage these potential costs, plans might choose not to cover spending on gene therapy, or impose restrictive policies beyond our assumptions to limit the number of patients who gain access to treatment [8]. Some plans have already warned they may not be able or willing to absorb the additional spending should a greater number of people become eligible for expensive gene therapy treatments once new ones reach the market [12].

Several innovative methods to finance gene therapy treatments complementary to the existing U.S. system have been proposed. The observation has been made that the empirical evidence supporting the effectiveness and durability of gene therapy is currently limited. Consequently, the provision of ‘full price’ reimbursement for these therapies under a typical policy constitutes a significant risk of failure that is currently borne by plans and employers alike. These methods thus aim to reduce the risks held by plans and employers in financing access to these therapies under conditions of extreme uncertainty. As one example, the drug companies selling onasemnogene abeparvovec-xioi and voretigene neparvovec offer outcome-based payments in which the company is only reimbursed (or paid a portion of full reimbursement) if the patients achieve predefined outcomes after treatment [34]. Mortgage-like payments and performance-based annuity payments are additional alternative ways to finance gene therapy treatments [35]. In September 2019, Cigna, one of the largest U.S. health insurance companies, announced a program called Embarc Benefit Protection in which employers, health plans, and unions would pay a monthly per-member premium that would provide its members with access to covered gene therapies. As of the time of writing, onasemnogene abeparvovec-xioi and several other gene and cell therapies are covered through this program at no out-of-pocket costs to patients, if their physicians authorize treatment [36].

A more ambitious proposal involves creating a national or international gene therapy reinsurance company that would perform a similar function to Embarc, but one serving many primary health insurance providers. By allowing multiple primary insurers to cede the specific risk of gene therapy patients to the reinsurer, these risks can be diversified over a much larger pool of members, thus lowering the cost of capital. The capital required for such a reinsurer can be raised through securitization techniques as described in Montazerhodjat et al. [35], which simulated such a structure, and concluded that the returns to investors would be attractive under a broad range of assumptions. However, their simulations were not specifically calibrated for gene therapy. Our framework may provide a useful complement to their analysis.

The reinsurer, on assuming the responsibility of delivering the gene therapies, may find it more cost-effective and produce higher quality outcomes to maintain nationally distributed gene therapy Centers of Excellence (CoEs). This function may seem too far afield for a reinsurance company, but the ability to have direct control over the quality of therapeutic delivery and to collect data on the performance of these therapies over time are two compelling reasons for the reinsurer to perform this task. The data collected from these centres will be critical for assessing the actuarial risk of reinsurance and for implementing performance-related contractual agreements, e.g., if a gene therapy ceases to be effective, then any remaining payments for the therapy will be cancelled.

An additional benefit of a single reinsurer to manage the risk and responsibility of delivering gene therapy is the ability of that reinsurer to avoid the adverse selection problem that often plagues individual insurers [37]. This problem arises when some insurers are willing to pay for gene therapy treatments while others are not, leading patients who require gene therapies to enrol en masse with those insurers providing coverage. Since these policies will likely have higher premiums to cover the high cost of gene therapy, patients have an incentive to leave the policy after receiving the treatment, leaving the insurers to pay the remaining cost without being able to recover the expenses. If a single reinsurer can aggregate this risk across a large pool of gene therapy patients and coordinate payouts across all insurers, this adverse selection problem may be greatly mitigated or altogether avoided. The viability of such a reinsurance vehicle depends critically on the various parameters of the modules in our simulation. In February 2023, the Biden Administration announced the Cell and Gene Therapy Access Model, in which state Medicaid agencies would assign the Centers for Medicare and Medicaid Services the responsibility to coordinate and administer multi-state contracts with manufacturers for certain gene therapies. This arrangement is effectively a type of reinsurance model in which the federal government ensures access to selected gene therapies with state Medicaid beneficiaries [38].

Returning to the limitations of our study, our simulation model requires multiple inputs that are inherently uncertain due to the lack of supporting empirical evidence. In this paper, we make multiple forecasting assumptions based on our best currently available knowledge, and we have tried to ensure they are methodologically conservative given the information to inform them. We have also made certain assumptions to simplify data collection and to make the computations tractable. We have provided tests of these assumptions in our sensitivity analyses. One great advantage of our simulation framework is that it can easily accept new data and new assumptions as the available information evolves. Finally, we excluded gene therapy clinical trials conducted solely outside the U.S., to be consistent with U.S. law governing FDA approvals. There are other reasons to exclude these trials from our analyses, including that their listing may not provide enough information to evaluate their consistency with the trials registered in the datasets we relied on to construct the sample of gene therapy trials. These exclusions will render our spending estimates conservative if and only if they target diseases and populations that are not targeted by the trials registered in the U.S. that met our inclusion criteria. If they are trials that are focused on the same diseases and populations that are targeted by U.S. registered trials, their existence will have no material impact on our results.

## Conclusion

In this paper, we developed and implemented a novel mathematical model to estimate the expected annual number of patients treated with gene therapy over time, and the annual cost of gene therapy in the U.S. overall and by payer. It is our hope that this study, and our estimates of the potential financial impact of gene therapy in the U.S., will provide more clarity on the potential clinical and fiscal impacts of this new class of treatment and identify uncertainties involved in any projection of expected spending on these therapies in aggregate and by specific U.S. payers. We hope this work will help decision-makers, including patients, physicians, hospital administrators, health plans, employers, drug companies and other innovators, and policymakers make informed decisions about future access and reimbursement for this novel therapeutic class.

### Supplementary information

Supplementary appendix

## Data Availability

The data used in this study are available by request from the authors.
