# Supplementary material for: The estimated annual financial impact of gene therapy in the United States
Source: Gene Ther. 2023 Nov 8;30(10-11):761–73. doi: 10.1038/s41434-023-00419-9 (PMC10678302; doi:10.1038/s41434-023-00419-9)
Supplement: Supplementary file 1 — Supplementary appendix [file 41434_2023_419_MOESM1_ESM.docx]

# SUPPLEMENTARY APPENDIX

# TABLE A1 Current Gene Therapy Clinical Trials

We list the clinical trials that are used in this study.

Table A1: List of clinical trials used in this study. ‘TT’ and ‘CT’ indicates ‘TrialTrove’ and ‘*clinicaltrials.gov*’ respectively.

| **Trial Title** | **Disease** | **Sponsors** | **Source** |
| --- | --- | --- | --- |
| Randomized, double-blind, placebo-controlled study of AMG0(HGF plasmid) for patients with arteriosclerosis obliterans | Arteriosclerosis Obliterans | AnGes | TT |
| Tisagenlecleucel Versus Standard of Care in Adult Patients With Relapsed or Refractory Aggressive B-cell Non-Hodgkin Lymphoma: A Randomized, Open Label,  Phase III Trial (BELINDA) | B-Cell Non-Hodgkin’s  Lymphoma | Novartis | TT |
| A Global Randomized Multicenter Phase III Trial of JCAR017 Compared to Standard of Care in Adult Subjects With High-risk, Second-line, Transplant-eligible Relapsed or Refractory Aggressive B-cell Non-Hodgkin Lymphomas (TRANSFORM). | B-Cell Non-Hodgkin’s  Lymphoma | Celgene | TT |
| A Phase III, Open Label Study to Evaluate the Safety and Efficacy of INSTILADRIN (rAd-IFN)/Syn3) Administered Intravesically to Patients With High Grade, BCG Unresponsive Non-Muscle Invasive Bladder Cancer (NMIBC) | BCG Unresponsive  NMIBC | FKD Therapeutics | TT |
| A Phase III Study of BC-819 in Patients with Bladder Cancer who Failed Initial Treatment of BCG | BCG Unresponsive  NMIBC | Anchiano Therapeutics | TT |

|  |  |  |
| --- | --- | --- |

| **Trial Title** | **Disease** | **Sponsors** | **Source** |
| --- | --- | --- | --- |
| A Phase 3 Single Arm Study Evaluating the Efficacy and Safety of Gene Therapy in Subjects With Transfusion-dependent beta-Thalassemia, Who do Not Have a beta0/beta0 Genotype, by Transplantation of Autologous CD34+ Stem Cells Transduced Ex Vivo With a Lentiviral betaA-T87Q-Globin Vector in Subjects *<* or = 50 Years of Age | Beta-Thalassemia | bluebird bio | TT |
| A Phase 3 Single Arm Study Evaluating the Efficacy and Safety of Gene Therapy in Subjects With Transfusiondependent beta-Thalassemia, Who Have a beta0/beta0 Genotype, by Transplantation of Autologous CD34+ Stem Cells Transduced Ex Vivo With a Lentiviral betaA-T87Q-Globin Vector in Subjects *<* or = 50 Years of Age | Beta-Thalassemia | bluebird bio | TT |
| An Integrated Phase II/III, Open Label, Randomized and Controlled Study of the Safety and Efficacy of CG0070 Adenovirus Vector Expressing GM-CSF in Patients With Non-Muscle Invasive Bladder Cancer With Carcinoma In Situ Disease Who Have Failed BCG Bladder Oncolytic virus for Non-muscle invasive bladder cancer Disease (BOND) | Bladder Cancer, in situ concurrent with Papillary Tumors | Cold Genesys | TT |
| A Phase 2/3 Study of the Efficacy and Safety of Hematopoietic Stem Cells Transduced With LentiD Lentiviral Vector for the Treatment of Cerebral  Adrenoleukodystrophy (CALD) | Cerebral  Adrenoleukodystrophy  (CALD) | bluebird bio | CT |
| Efficacy and Safety of AAV2-REP1 for the Treatment of Choroideremia | Choroideremia | Nightstar Therapeutics | CT |

| **Trial Title** | **Disease** | **Sponsors** | **Source** |
| --- | --- | --- | --- |
| A Phase 3 Double-Blind, Randomized, PlaceboControlled Study to Evaluate the Safety and Efficacy of AMG0 in Subjects With Critical Limb Ischemia Efficacy and Safety of AMG0 in Subjects With Critical  Limb Ischemia (AGILITY) | Critical Limb Ischemia | AnGes | TT |
| Safety and Efficacy of Recombinant Adeno-Associated Virus Containing the CFTR Gene in the Treatment of Cystic Fibrosis | Cystic Fibrosis | Targeted Genetics Corporation/ Cystic  Fibrosis Foundation  Therapeutics | CT |
| A Placebo Controlled, Double-blind, Randomized, Parallel-group, Multi-center Phase III study to determine the Efficacy and Safety of TisssueGene-C in Patients with Degenerative Arthritis | Degenerative Arthritis | Kolon Life Science | TT |
| Safety and Efficacy Study of Pl-VEGF165 to Treat Diabetic Foot Syndrome | Diabetic Foot Syndrome | Human Stem Cells  Institute | TT |
| A Phase III, Double-blind, Randomized, Placebocontrolled, Multicenter Study to Asses the Safety and Efficacy of VM202 to Treat Chronic Nonhealing Foot Ulcers in Diabetic Patients With Concomitant Peripheral Arterial Disease (PAD) | Diabetic Foot Ulcers | Helixmith | TT |
| A Phase III, Double-Blind, Randomized, Placebo-  Controlled, Multicenter Study to Assess the Safety and Efficacy of VM202 in Subjects With Painful Diabetic Peripheral Neuropathy | Diabetic Peripheral Neuropathy | Helixmith | TT |
| A Phase III, Randomized, Open-Label Study Evaluating Efficacy of Axicabtagene Ciloleucel Versus Standard of Care Therapy in Subjects With Relapsed/Refractory Diffuse Large B Cell Lymphoma | Diffuse Large B Cell Lymphoma (DLBCL) | Gilead Sciences/Kite  Pharma | TT |

| **Trial Title** | **Disease** | **Sponsors** | **Source** |
| --- | --- | --- | --- |
| A Multi-center Phase III, Randomized, Open-Label Trial of Vigil (Bi-shRNAfurin and GMCSF Augmented Autologous Tumor Cell Immunotherapy) in Combination With Irinotecan and Temozolomide as a SecondLine Regimen for Ewing’s Sarcoma | Ewing’s Sarcoma | Gradalis | TT |
| A Phase III Study of INGN 241 in Combination with Radiation Therapy in Patients with Advanced Solid Tumors and Head and neck cancer. | Head and Neck Cancer | Introgen Therapeutics | TT |
| An Open-Label, Randomized, Multi-Center Phase III  Clinical Trial Comparing E10A Plus Chemotherapy And Chemotherapy Alone For Treatment Of Head And Neck Cancer | Head and Neck Cancer | Marsala Biotech | TT |
| A Randomized, Open-label, Multi-center Phase III Study Designed to Evaluate the Safety and Efficacy of E10A in Patients With Recurrent/Unresectable Squamous Cell Carcinoma of the Head and Neck Region | Head and Neck Cancer | Guangzhou Double Bioproducts Co. | TT |
| A Phase III, Pivotal, Randomized, Placebo-controlled, Double-Blind, Multicenter Study to Evaluate RT-100 AC6 Gene Transfer in Patients with Heart Failure and Reduced Left Ventricular Ejection Fraction;Heart Failure with Reduced Left Ventricular Ejection Fraction: One-time Gene Transfer Using RT-100 Intracoronary Administration of Adenovirus 5 encoding Human AC6 (FLOURISH) | Heart Failure | Renova Therapeutics | TT |

| **Trial Title** | **Disease** | **Sponsors** | **Source** |
| --- | --- | --- | --- |
| A Phase 3 Open-Label, Single-Arm Study To Evaluate The Efficacy and Safety of BMN 270, an AdenoAssociated Virus Vector-Mediated Gene Transfer of Human Factor VIII in Hemophilia A Patients With Residual FVIII Levels = 1 IU/dL Receiving Prophylactic FVIII Infusions | Hemophilia A | BioMarin | TT |
| Phase 3 Study To Evaluate Efficacy/Safety of Valoctocogene Roxaparvovec an AAV Vector-Mediated Gene Transfer of hFVIII at a Dose of 4E13vg/kg in Hemophilia A Patients With Residual FVIII Levels *<* or = 1IU/dL Receiving Prophylactic FVIII Infusions | Hemophilia A | BioMarin | TT |
| A Phase III Run In trial to Evaluate SPK-8011 in Patients with Hemophilia A | Hemophilia A | Roche/Spark Therapeutics | TT |
| An open-label, single-dose, multi-center, multi-national, Phase III pivotal trial to investigate efficacy and safety of AMT-061 in severe or moderately severe hemophilia B; HOPE-B: Trial of AMT-061 in Severe or Moderately Severe Hemophilia B Patients; Phase III, Open-label, Single-dose, Multi-center, Multinational Trial Investigating a Serotype 5 Adeno-associated Viral Vector Containing the Padua Variant of a Codon-optimized Human Factor IX Gene (AAV5-hFIXco-Padua, AMT-061) Administered to Adult Subjects With Severe or Moderately Severe Hemophilia B | Hemophilia B | uniQure | TT |

| **Trial Title** | **Disease** | **Sponsors** | **Source** |
| --- | --- | --- | --- |
| A Pivotal Phase III Study of PF-06838435 in Patients with Hemophilia B; Phase 3, Open Label, Single Arm Study To Evaluate Efficacy And Safety Of Fix Gene Transfer With Pf-06838435 (Raav-Spark100-HfixPadua) In Adult Male Participants With Moderately  Severe To Severe Hemophilia B (Fix:C *<* or =2%) | Hemophilia B | Pfizer | TT |
| A Pivotal Phase III Study to Evalaute AMT-060 in Patients with Hemophilia B | Hemophilia B | uniQure | TT |
| Multicenter Randomized Controlled Trial of Adenovirus-mediated Adjuvant Gene Therapy Improving Outcome of Liver Transplantation in Patients With Advanced Hepatocellular Carcinoma | Hepatocellular Carcinoma | Wuhan Tiandakang  Bio-Tech Engineering Co./ Shenzhen Tiandakang Gene Engineering Co. | TT |
| A Phase III Randomized, Open-Label Study Comparing Pexa Vec (Vaccinia GM CSF / Thymidine KinaseDeactivated Virus) Followed by Sorafenib Versus Sorafenib in Patients With Advanced Hepatocellular Carcinoma (HCC) Without Prior Systemic Therapy | Hepatocellular Carcinoma | Transgene/ Sillajen  Biotherapeutics /Jennerex/  Lees Pharmaceutical | TT |
| Phase III Prospective, Open-Label, Parallel-Group, Randomized, Multicenter Trial Comparing the Efficacy of Surgery, Radiation, and Injection of Murine Cells Producing Herpes Simplex Thymidine Kinase Vector Followed by Intravenous Ganciclovir Against the Efficacy of Surgery and Radiation in the Treatment of Newly Diagnosed, Previously Untreated Glioblastoma Multiforme | High-Grade Glioma | Novartis/Sandoz | TT |

| **Trial Title** | **Disease** | **Sponsors** | **Source** |
| --- | --- | --- | --- |
| A Controlled, Randomised, Parallel Group Study Of The Efficacy And Safety Of Herpes Simplex Virus Thymidine Kinase Gene Therapy (Cerepro) with Subsequent Ganciclovir For The Treatment Of Patients With Operable High-Grade Glioma. | High-Grade Glioma | Trizell | TT |
| A Randomized, Double-Blind, Placebo-Controlled, Multi-Center, Phase 3 Study to Determine the Efficacy of TG-C in Subjects With Kellgren and Lawrence Grade (KLG) 2 or 3 Osteoarthritis of the Knee | Knee Osteoarthritis with Kellgren & Lawrence Grade 2 or 3 | Kolon TissueGene | TT |
| A Multicenter, Randomized, Placebo Controlled, Double-blind, Parallel, Phase III Clinical Trial to Evaluate the Efficacy and Safety of Invossa K Injection in Patients Diagnosed as Knee Osteoarthritis With Kellgren & Lawrence Grade 2 | Knee Osteoarthritis with Kellgren & Lawrence Grade 2 or 3 | Kolon Life Science | TT |
| Safety and Efficacy Study in Subjects With Leber Congenital Amaurosis | Leber Congenital  Amaurosis due to  RPE65 Mutations | Spark Therapeutics | CT |
| Efficacy Study of GS010 for Treatment of Vision Loss From 7 Months to 1 Year From Onset in LHON Due to the ND4 Mutation | Leber Hereditary Optic Neuropathy | GenSight Biologics | CT |
| Efficacy Study of GS010 for the Treatment of Vision Loss up to 6 Months From Onset in LHON Due to the ND4 Mutation | Leber Hereditary Optic Neuropathy | GenSight Biologics | CT |
| Efficacy and Safety Study of Bilateral Intravitreal Injection of GS010 for the Treatment of Vision Loss up to 1 Year From Onset in LHON Due to the ND4 Mutation | Leber Hereditary Optic Neuropathy | GenSight Biologics | CT |

| **Trial Title** | **Disease** | **Sponsors** | **Source** |
| --- | --- | --- | --- |
| Tisagenlecleucel Versus Blinatumomab or Inotuzumab for Adult Patients With Relapsed/Refractory B-cell Precursor Acute Lymphoblastic Leukemia: A Randomized Open Label, Multicenter, Phase III Trial | Leukemia (Acute Lymphoblastic) | Novartis | TT |
| Phase IIIb Study for Relapsed/Refractory Pediatric/Young Adult Acute Lymphoblastic Leukemia Patients to be Treated With CTL019 | Leukemia (Acute Lymphoblastic) | Novartis | TT |
| A Phase II/III Prospective, Open Label Study to Evaluate Safety and Efficacy of Intravenous Autologous CD19 CAR-T Cells for Relapsed/ Refractory B-Acute Lymphoblastic Leukemia | Leukemia (Acute Lymphoblastic) | Gaia Science | TT |
| A Randomized Phase II/III Study of *αβ* T Cell-  Depleted, Related, Haploidentical Hematopoietic Stem Cell Transplant (Haplo-HSCT) Plus Rivogenlecleucel vs. Haplo-HSCT Plus Post-Transplant Cyclophosphamide (PTCy) in Patients With AML or MDS | Leukemia (Acute Myelogenous) | Bellicum Pharmaceuticals | TT |
| Randomized, Registrational, Controlled Study of BPX501 with Allogeneic Hematopoietic Stem Cells (Allo-  HSCT) in Patients with Acute Myelogenous Leukemia | Leukemia (Acute Myelogenous) | Bellicum Pharmaceuticals | TT |
| TK008: Randomized Phase III Trial of Haploidentical HCT With or Without an Add Back Strategy of HSVTk Donor Lymphocytes in Patients With High Risk Acute Leukemia | Leukemia (Acute Myelogenous) | Molmed | TT |
| A Phase IIb/III Study of AST-VAC1 in Patients with Acute Myelogenous Leukemia (AML) | Leukemia (Acute Myelogenous) | Asterias/Lineage Cell Therapeutics | TT |
| A Study of Glybera for the Treatment of Lipoprotein Lipase (LPL) Deficiency | Lipoprotein Lipase Deficiency (LPLD) | uniQure | TT |

| **Trial Title** | **Disease** | **Sponsors** | **Source** |
| --- | --- | --- | --- |
| A Study to Determine the Safety and Efficacy in Lipoprotein Lipase-Deficient Subjects After Intramuscular Administration of AMT-011, an AdenoAssociated Viral Vector Expressing Human Lipoprotein LipaseS447X. | Lipoprotein Lipase Deficiency (LPLD) | uniQure | TT |
| An Open-label Study to Assess the Efficacy and Safety of Alipogene Tiparvovec (AMT-011), Human LPL [S447X], Expressed by an Adeno-Associated Viral Vector After Intramuscular Administration in LPL-deficient Adult Subjects | Lipoprotein Lipase Deficiency (LPLD) | uniQure | TT |
| A Study of AMT-011 in Patients With LPL Deficiency | Lipoprotein Lipase Deficiency (LPLD) | uniQure | TT |
| A Phase III Trial of Glybera for Dyslipidemia | Lipoprotein Lipase  Deficiency (LPLD) | uniQure | TT |
| Phase II/III study of Ad-IFNg in Cutaneous T-cell lymphoma | Lymphoma | Transgene | TT |
| A Safety and Efficacy Study of Cryopreserved GSK2696274 for Treatment of Metachromatic Leukodystrophy (MLD) | Metachromatic Leukodystrophy | GlaxoSmithKline | CT |
| PV-10 Intralesional Injection vs Systemic Chemotherapy or Oncolytic Viral Therapy for Treatment of Locally Advanced Cutaneous Melanoma | Melanoma (Locally Advanced Cutaneous) | Provectus  Biopharmaceuticals | TT |
| A Phase Ib/III, Multicenter, Trial of Talimogene Laherparepvec in Combination With Pembrolizumab (MK-3475) for Treatment of Unresectable Stage IIIB to IVM1c Melanoma (MASTERKEY-265/KEYNOTE-  034) | Melanoma  (Metastatic) | Amgen/ Merck &  Co./Merck Sharp & Dohme  (MSD) | TT |

| **Trial Title** | **Disease** | **Sponsors** | **Source** |
| --- | --- | --- | --- |
| A Phase III Clinical Trial to Evaluate the Safety and Efficacy of Treatment With 2 mg Intralesional Allovectin-7 Compared to Dacarbazine (DTIC) or Temozolomide (TMZ) in Subjects With Recurrent Metastatic Melanoma;Allovectin-7 Immunotherapeutic for Metastatic Melanoma (AIMM). | Melanoma  (Metastatic) | Brickell Biotech, AnGes | TT |
| A Randomized Phase III Clinical Trial to Evaluate the Efficacy and Safety of Treatment With OncoVEXGMCSF Compared to Subcutaneously Administered GMCSF in Melanoma Patients With Unresectable Stage IIIb, IIIc and IV Disease | Melanoma  (Metastatic) | Amgen | TT |
| An Extension Protocol to Evaluate the Efficacy and Safety of Extended Use Treatment With OncoVEXGMCSF for Eligible Melanoma Patients Participating in Study 005/05 | Melanoma  (Metastatic) | Amgen | TT |
| A Controlled, Randomized Phase III Trial Comparing the Response to Dacarbazine With and Without Allovectin-7 in Patients With Metastatic Melanoma. | Melanoma  (Metastatic) | Brickell Biotech | TT |
| Open-label, Single-arm, Multi-center Study of Intracerebral Administration of Adeno-associated Viral (AAV) Serotype rh.10 Carrying Human N-sulfoglucosamine Sulfohydrolase (SGSH) cDNA for Treatment of Mucopolysaccharidosis Type IIIA | Mucopolysaccharidosis Type IIIa | LYSOGENE | TT |

| **Trial Title** | **Disease** | **Sponsors** | **Source** |
| --- | --- | --- | --- |
| A Phase III, Single Arm, Multi-Center Study to Assess the Efficacy and Safety of Clarithromycin(Biaxin)Lenalidomide-Low-Dose-Dexamethasone (BiRd) Combined With B-cell Muturation Antigen (BCMA)Directed Chimeric Antigen Receptor (CAR) T-cell Therapy in Patients With Newly Diagnosed Multiple Myeloma | Multiple Myeloma (Newly Diagnosed) | Shanghai Unicar-Therapy Bio-medicine | TT |
| Clinical Trial of Recombinant Adenovirus-p53 (Gendicine) Combined with Radiotherapy in Nasopharyngeal Carcinoma Patients.; | Nasopharyngeal Carcinoma | Shenzhen SiBiono GeneTech Co. | TT |
| A Phase II/III, Multi-Center, Open-Label, Randomized Study to Compare the Effectiveness and Safety of Intralesional Administration of RPR/INGN 201 in Combination with Taxotere and Carboplatin and Radiotherapy Versus Taxotere and Carboplatin and Radiotherapy Alone in Patients with Locally Advanced Unresectable  Non-Small Cell Lung Cancer (NSCLC) | NSCLC | Introgen Therapeutics | TT |
| A Phase IIB/III Randomized, Double-blind, Placebo Controlled Study Comparing First Line Therapy With or Without TG4010 Immunotherapy Product in Patients With Stage IV Non-Small Cell Lung Cancer (NSCLC) | NSCLC | Transgene | TT |
| Phase III multi-center, open, randomized clinical trial of percutaneous intratumoral injection of genetically engineered adenovirus (injection of H101), IL-2, TB hyperthermia and systemic chemotherapy in the treatment of advanced non-small cell lung cancer | NSCLC | Shanghai Sunway Biotech | TT |

| **Trial Title** | **Disease** | **Sponsors** | **Source** |
| --- | --- | --- | --- |
| Phase III Study of Lucanix (Belagenpumatucel-L) in Advanced Non-small Cell Lung Cancer: An International Multicenter, Randomized, Double-blinded, Placebo-controlled Study of Lucanix Maintenance Therapy for Stages III/IV NSCLC Subjects Who Have Responded to or Have Stable Disease Following One Regimen of Front-line, Platinum-based Combination Chemotherapy; Survival, Tumor-free, Overall and Progression-free (STOP) | NSCLC Stage 3 | Activate Immunotherapy | TT |
| rAd-p53 Combined Chemotherapy Via Selective Arterial Cannula in The Treatment of Advanced Oral Cancer, A Randomized Controlled Trial | Oral Cancer (Advanced) | Shenzhen SiBiono GeneTech Co. | TT |
| A Randomized, Controlled, Double-Arm, Open-Label,  Multi-Center Study of Ofranergene Obadenovec (VB111) Combined With Paclitaxel vs. Paclitaxel  Monotherapy for the Treatment of Recurrent PlatinumResistant Ovarian Cancer | Ovarian Cancer  (Platinum-Resistant) | Gynecologic Oncology  Group (GOG)/ VBL  Therapeutics | TT |
| A Phase II/III Trial of Chemotherapy Alone Versus Chemotherapy Plus SCH 58500 in Newly Diagnosed Stage III Ovarian and Primary Peritoneal Cancer Patients With Greater Than or Equal to 0.5 cm and Less Than or Equal to 2 cm Residual Disease Following  Surgery | Ovarian Cancer,  Primary Peritoneal  Cavity Cancer | Merck & Co./Merck Sharp & Dohme (MSD) | TT |
| A Randomized, Phase II/III, Study of TNFerade Biologic With 5-FU and Radiation Therapy for First-Line Treatment of Unresectable Locally Advanced Pancreatic Cancer | Pancreatic Cancer (Locally Advanced) | Precigen | TT |

| **Trial Title** | **Disease** | **Sponsors** | **Source** |
| --- | --- | --- | --- |
| Phase II/III Study of ProSavin for the Treatment of  Parkinson’s Disease | Parkinson’s Disease | Oxford BioMedica | TT |
| Phase III Trial of CERE-120 for Parkinson’s Disease | Parkinson’s Disease | Sanofi/Sanofi Genzyme, Sangamo Therapeutics | TT |
| A Randomized, Placebo-controlled Phase IIIa Pivotal Confirmatory Study to Evaluate Safety and Efficacy of VY-AADC in Patients with Parkinson’s Disease | Parkinson’s Disease | Neurocrine Biosciences | TT |
| A Randomized Double-Blind Placebo-Controlled Parallel Group Study of the Efficacy and Safety of XRP0038/NV1FGF on Amputation or Any Death in  Critical Limb Ischemia Patients With Skin Lesions | Peripheral Artery Disease | Sanofi | TT |
| Efficiency, Safety and Portability of Neovasculgen | Peripheral Artery Disease | Human Stem Cell  Institute, Russia | CT |
| Gene Therapy using Intramuscular Administration of  AMG0001 in Patients with Peripheral Arterial Disease; | Peripheral Artery Disease | AnGes | TT |
| Hepatocyte Growth Factor to Improve Functioning in Peripheral Artery Disease: The HI-PAD Study; | Peripheral Artery Disease | Helixmith | TT |
| A phase III study of HGF Plasmid in Peripheral Arterial Disease (PAD) in the US | Peripheral Artery Disease | AnGes | TT |
| Phase 3 Study of Efficiency, Safety and Portability of Gene Therapy Drug Neovasculgen (DNA Encoding the 165-amino-acid Isoform of Human Vascular Endothelial Growth Factor (pCMV - VEGF165) for Peripheral Arterial Disease Complex Treatment | Peripheral Artery Disease | Human Stem Cells  Institute | TT |
| Provenge (Sipuleucel-T) Active Cellular Immunotherapy Treatment of Metastatic Prostate Cancer After Failing Hormone Therapy | Prostate Cancer | Dendreon | CT |

| **Trial Title** | **Disease** | **Sponsors** | **Source** |
| --- | --- | --- | --- |
| A Randomized, Controlled Trial of ReplicationCompetent Adenovirus-Mediated Suicide Gene Therapy in Combination With IMRT Versus IMRT Alone for the Treatment of Newly-Diagnosed Prostate Cancer With an Intermediate Risk Profile | Prostate Cancer (Localized) | Henry Ford Health System | TT |
| A Randomized Controlled Trial of ProstAtak as Adjuvant to Up-front Radiation Therapy For Localized Prostate Cancer | Prostate Cancer (Localized) | Candel Therapeutics | TT |
| A Phase III Randomized, Open-Label Study of CG1940 and CG8711 Versus Docetaxel and Estramustine in Patients with Metastatic Hormone-Refractory Prostate Cancer Who are Chemotherapy-Naive. | Prostate Cancer  (Metastatic  Hormone-Refractory) | ANI Pharmaceuticals, Takeda | TT |
| A Phase III Randomized, Open-Label Study of CG1940 and CG8711 Versus Docetaxel and Prednisone in Patients With Metastatic Hormone-Refractory Prostate Cancer Who Are Chemotherapy-Naive. | Prostate Cancer  (Metastatic  Hormone-Refractory) | ANI Pharmaceuticals, Takeda | TT |
| A Phase III Randomized, Open-Label Study of Docetaxel in Combination With CG1940 and CG8711 Versus Docetaxel and Prednisone in Taxane-Nave Patients With Metastatic Hormone-Refractory Prostate Cancer With Pain. | Prostate Cancer  (Metastatic  Hormone-Refractory) | ANI Pharmaceuticals, Takeda | TT |
| A Randomized Controlled Trial Of AdV-tk + Valacyclovir Administered During Active Surveillance For Newly Diagnosed Prostate Cancer | Prostate Cancer (Newly Diagnosed) | Candel Therapeutics | TT |
| An Open label,Randomized, Multi-Centered, IntraPatient Controlled Phase III Study of FCX-007 in Patients with Recessive Dystrophic Epidermolysis Bullosa (RDEB) | Recessive Dystrophic Epidermolysis Bullosa | Fibrocell Science | TT |

| **Trial Title** | **Disease** | **Sponsors** | **Source** |
| --- | --- | --- | --- |
| VITAL: A Pivotal Phase 3 Study of EB-101 for the Treatment of Recessive Dystrophic Epidermolysis Bullosa (RDEB) (GENE TRANSFER) | Recessive Dystrophic Epidermolysis Bullosa | Stanford University  Medical Center/ Abeona  Therapeutics | TT |
| A Phase III, Randomized, Controlled, Double-Arm, Open-Label, Multi-center Study of VB-111 Combined  With Bevacizumab vs. Bevacizumab Monotherapy in  Patients With Recurrent Glioblastoma | Recurrent  Glioblastoma | VBL Therapeutics | TT |
| A Phase II/III Randomized, Open-Label Study of Toca 511, a Retroviral Replicating Vector, Combined With Toca FC Versus Standard of Care in Subjects Undergoing Planned Resection for Recurrent Glioblastoma or Anaplastic Astrocytoma | Recurrent  Glioblastoma | Tocagen | TT |
| A Randomized, Double-Blind, Placebo-Controlled, Parallel Group, Multicenter, Phase 3 Study to Evaluate the Safety and Efficacy of Ad5FGF-4 in Patients With Refractory Angina Due to Myocardial Ischemia;Ad5FGF-4 In Patients With Refractory Angina Due to Myocardial Ischemia (AFFIRM) | Refractory Angina due to Myocardial Ischemia (AFFIRM) | Gene Biotherapeutics/Angionetics | TT |
| A Phase III, Multicenter, Randomized, Open-label Study to Compare the Efficacy and Safety of bb2121 Versus Standard Triplet Regimens in Subjects With Relapsed and Refractory Multiple Myeloma (RRMM) (KarMMa-3) | Relapsed and  Refractory Multiple  Myeloma (RRMM) | Celgene | TT |
| A Single Global Phase 3 trial of RST-001 in Patients With Retinitis Pigmentosa (RP) | Retinitis Pigmentosa | Abbvie/Allergan | TT |
| A Phase II/III Expansion Study to Evaluate Safety and Efficacy of NSR-RPGR in Patients with a Diagnosis of X  - Linked Retinitis Pigmentosa due to RPGR mutations | Retinitis Pigmentosa | NightstaRx | TT |

| **Trial Title** | **Disease** | **Sponsors** | **Source** |
| --- | --- | --- | --- |
| Phase 3 HGB-210 study of LentiGlobin in patients with  SCD | Sickle Cell Anemia | bluebird bio | TT |
| Open-label, historical controlled study of AVXS-101 for treatment of spinal muscular atrophy | Spinal Muscular Atrophy | Novartis/AveXis | TT |
| A Multi-National Study of a One-Time Intrathecal Dose of AVXS-101 in Patients with Spinal Muscular Atrophy Types 1, 2, 3 | Spinal Muscular Atrophy | Novartis/AveXis | TT |
| A Global Study of a Single, One-Time Dose of AVXS101 Delivered to Infants With Genetically Diagnosed and Pre-symptomatic Spinal Muscular Atrophy With Multiple Copies of SMN2 | Spinal Muscular Atrophy Type 1 | Novartis/AveXis | TT |
| European, Phase 3, Open-Label, Single-Arm, SingleDose Gene Replacement Therapy Clinical Trial for Patients With Spinal Muscular Atrophy Type 1 With One or Two SMN2 Copies Delivering AVXS-101 by Intravenous Infusion | Spinal Muscular Atrophy Type 1 | Novartis/AveXis | TT |
| Phase 3, Open-Label, Single-Arm, Single-Dose Gene Replacement Therapy Clinical Trial for Patients With Spinal Muscular Atrophy Type 1 With One or Two SMN2 Copies Delivering AVXS-101 by Intravenous Infusion | Spinal Muscular Atrophy Type 1 | Novartis/AveXis | TT |
| A Phase Ib/III Multicenter, Randomized, Trial of Talimogene Laherparepvec in Combination With Pembrolizumab for the Treatment of Subjects With Recurrent or Metastatic Squamous Cell Carcinoma of the Head and Neck | Squamous Cell Cancer of Head and Neck or  Esophagus | Amgen/ Merck &  Co./Merck Sharp & Dohme  (MSD) | TT |

| **Trial Title** | **Disease** | **Sponsors** | **Source** |
| --- | --- | --- | --- |
| Phase III randomized clinical trial of intratumoral injection of E1B gene-deleted adenovirus (H101) combined with cisplatin-based chemotherapy in treating squamous cell cancer of head and neck or esophagus. | Squamous Cell Cancer of Head and Neck or Esophagus | Shanghai Sunway Biotech | TT |
| Phase III Randomized Study of Ad5CMV-p53 Gene  Therapy (INGN 201) Versus Methotrexate in Patients With Refractory Squamous Cell Carcinoma of the Head and Neck (T301). | Squamous Cell Cancer of Head and Neck or Esophagus | Sanofi, Introgen Therapeutics | TT |
| A Phase III, Multi-Center, Open-Label, Randomized Study to Compare the Effectiveness and Safety of Intratumoral Administration of INGN 201 in Combination with Chemotherapy Versus Chemotherapy Alone in Patients with Squamous Cell Carcinoma of the Head and Neck (SCCHN) | Squamous Cell Cancer of Head and Neck or  Esophagus | Introgen Therapeutics | TT |
| A Randomized, Controlled, Parallel Group, Multicenter Phase 3 Study to Evaluate the Efficacy and Safety of Ad5FGF-4 Using SPECT Myocardial Perfusion Imaging in Patients With Stable Angina Pectoris | Stable Angina | Gene Biotherapeutics/  Angionetics/ Gene  Biotherapeutics | TT |
| A Randomized, Double Blind, Placebo Controlled, Parallel Group, Multicenter Study to Evaluate the Efficacy and Safety of Ad5FGF-4 in Female Patients With Stable Angina Pectoris Who Are Not Candidates for Revascularization;Angiogenesis in Women with Angina pectoris who are not candidates for Revascularization [AWARE] | Stable Angina | Gene Biotherapeutics/  Angionetics/ Gene  Biotherapeutics | TT |

| **Trial Title** | **Disease** | **Sponsors** | **Source** |
| --- | --- | --- | --- |
| A Multinational Multicenter, Randomized, Double Blind, Placebo Controlled Study to Evaluate the Efficacy and Safety of Ad5FGF-4 in Patients With Stable Angina;(The Angiogenic Gene Therapy Trial - 4 [AGENT 4]). | Stable Angina | Bayer AG/Bayer  HealthCare, Gene  Biotherapeutics | TT |
| A Multicenter, Randomized, Double-Blind, Placebo Controlled Study to Evaluate the Efficacy and Safety of Ad5FGF-4 in Patients With Stable Angina (The Angiogeneic Gene Therapy Trial - 3 [AGENT 3]) | Stable Angina | Bayer AG/Bayer  HealthCare, Gene  Biotherapeutics | TT |
| Multicentre, Randomized,Double Blind, Placebo Controlled Trial of Myocardial Angiogenesis Using VEGF165, Intramyocardial Gene Delivery in Patients  With Severe Angina Pectoris | Stable Angina | Johnson & Johnson | TT |
| A Pivotal Study of NY-ESO-1 in Patients with Synovial Sarcoma including Myxoid Round Cell Liposarcoma | Synovial Sarcoma | GlaxoSmithKline/ AdaptImmune | TT |

# TABLE A2 Diseases with ongoing gene therapy trials and their associated therapeutic areas

| **Disease** | **Therapeutic Area** |
| --- | --- |
| **– General Conditions –**  Arteriosclerosis Obliterans | Cardiovascular |
| Critical Limb Ischemia | Cardiovascular |
| Degenerative Arthritis | Autoimmune/Inflammation |
| Diabetic Foot Symptoms | Metabolic/Endocrinology |
| Diabetic Foot Ulcers | Metabolic/Endocrinology |
| Diabetic Peripheral Neuropathy | Metabolic/Endocrinology |
| Heart Failure | Cardiovascular |
| Knee Osteoarthritis with Kellgren & Lawrence Grade 3 | Autoimmune/Inflammation |
| Parkinson’s Disease | CNS |
| Peripheral Artery Disease | Cardiovascular |
| Refractory Angina due to Myocardial Ischemia (AFFIRM) | Cardiovascular |
| Stable Angina | Cardiovascular |
| **– Rare Diseases –**  Beta-Thalassemia | Metabolic/Endocrinology |
| Cerebral Adrenoleukodystrophy (CALD) | CNS |
| Choroideremia | Ophthalmology |
| Cystic Fibrosis | Cardiovascular |
| Ewing’s Sarcoma | Oncology |
| Hemophilia A | Metabolic/Endocrinology |
| Hemophilia B | Metabolic/Endocrinology |
| Leber Congenital Amaurosis due to RPE65 Mutations | Ophthalmology |
| Leber Hereditary Optic Neuropathy | Ophthalmology |
| Lipoprotein Lipase Deficiency (LPLD) | Metabolic/Endocrinology |
| Metachromatic Leukodystrophy | Metabolic/Endocrinology |
| Mucopolysaccharidosis Type IIIa | CNS |
| Recessive Dystrophic Epidermolysis Bullosa | Autoimmune/Inflammation |
| Retinitis Pigmentosa | Ophthalmology |
| Sickle Cell Anemia | Metabolic/Endocrinology |
| Spinal Muscular Atrophy | CNS |
| Spinal Muscular Atrophy Type 1 | CNS |
| **– Cancer –**  B-Cell Non-Hodgkin’s Lymphoma | Oncology |
| BCG Unresponsive NMIBC | Oncology |
| Bladder Cancer, in situ concurrent with Papillary Tumors | Oncology |

Table A2 – continued from previous page

| **Disease** | **Therapeutic Area** |
| --- | --- |
| Diffuse Large B Cell Lymphoma (DLBCL) | Oncology |
| Head and Neck Cancer | Oncology |
| Hepatocellular Carcinoma | Oncology |
| High-Grade Glioma | Oncology |
| Leukemia (Acute Lymphoblastic) | Oncology |
| Leukemia (Acute Myelogenous) | Oncology |
| Lymphoma | Oncology |
| Melanoma (Locally Advanced Cutaneous) | Oncology |
| Melanoma (Metastatic) | Oncology |
| Multiple Myeloma (Newly Diagnosed) | Oncology |
| Nasopharyngeal Carcinoma | Oncology |
| NSCLC | Oncology |
| NSCLC Stage 3 | Oncology |
| Oral Cancer (Advanced) | Oncology |
| Ovarian Cancer (Platinum-Resistant) | Oncology |
| Ovarian Cancer, Primary Peritoneal Cavity Cancer | Oncology |
| Pancreatic Cancer (Locally Advanced) | Oncology |
| Prostate Cancer | Oncology |
| Prostate Cancer (Localized) | Oncology |
| Prostate Cancer (Metastatic Hormone-Refractory) | Oncology |
| Prostate Cancer (Newly Diagnosed) | Oncology |
| Recurrent Glioblastoma | Oncology |
| Relapsed and Refractory Multiple Myeloma (RRMM) | Oncology |
| Squamous Cell Cancer of Head and Neck or Esophagus | Oncology |
| Synovial Sarcoma | Oncology |

# TABLE A3 Number of current patients and annual new patients for each disease

| **Disease** | **Current patients** | **New patients per year** |
| --- | --- | --- |
| **– General Conditions –**  Arteriosclerosis Obliterans | [96]8500000 | *192100 |
| Critical Limb Ischemia | [86]975000 | [86]300000 |
| Degenerative Arthritis | [163]27000000 | *486000 |
| Diabetic Foot Ulcers | [132]2250000 | [131]112500 |
| Diabetic Peripheral Neuropathy | [69,97,170]9441480 | [69,97,170]467400 |
| Heart Failure | [108]5800000 | [108]812000 |
| Knee Osteoarthritis with Kellgren & Lawrence Grade 2 or/and 3 | *2929730 | [163]542000 |
| Parkinson’s Disease | [20]500000 | [20]50000 |
| Peripheral Artery Disease | [96]8500000 | *564400 |
| Refractory Angina due to Myocardial Ischemia (AFFIRM) | ^[5]^8200000 | [5,73]565000 |
| Stable Angina | ^[44]^10000000 | [44]500000 |
| **– Rare Diseases –**  Beta-Thalassemia | [11]1000 | [139]3277 |
| Cerebral Adrenoleukodystrophy (CALD) | [66]411 | [66]37 |
| Choroideremia | [129]6554 | [129]77 |
| Cystic Fibrosis | [65]30000 | [65]1000 |
| Ewing’s Sarcoma | [13,14,61]15003 | [13,14,61]200 |
| Hemophilia A | [47]16000 | [47]360 |
| Hemophilia B | [47]4000 | [47]90 |
| Leber Congenital Amaurosis due to RPE65 Mutations | [79]187 | [79]19 |
| Leber Hereditary Optic Neuropathy | [27]6540 | [27]654 |
| Lipoprotein Lipase Deficiency (LPLD) | [75]328 | *33 |
| Metachromatic Leukodystrophy | [48,141]9333 | [48,141]771 |
| Mucopolysaccharidosis Type IIIa | [30]1638 | [30]39 |
| Recessive Dystrophic Epidermolysis Bullosa | [115]100 | *10 |
|  | Continued on next page | |

We source the patient prevalence and incidence of the diseases from different sources. When necessary, we compute the prevalence from the incidence using Equation 1, or vice versa, using Equation 2. Our results are shown in Table A3. These numbers do not reflect the adjustments we make to NSC lung cancer, prostrate cancer and spinal muscular atrophy in order to minimize overlapping patient groups. An asterisk (∗) indicates that either the prevalence is computed from the incidence using Equation 1, or vice versa, using Equation 2.

| **Disease** | **Current New patients patients per year** | |
| --- | --- | --- |
| Retinitis Pigmentosa | [146]87387 [146]8739 | |
| Sickle Cell Anemia | [58]100000 [59]58745 | |
| Spinal Muscular Atrophy | [125]8526 [125]290 | |
| Spinal Muscular Atrophy Type 1 | [42]17500 [83]500 | |
| **– Cancer –**  B-Cell Non-Hodgkin’s Lymphoma | [9,10]694704 [9,10]74200 | |
| BCG Unresponsive NMIBC | [12,109,120,121]371933[12,109,120,121]42625 | |
| Bladder Cancer, in situ concurrent with Papillary Tumors | [12]356720 | [12]41040 |
| Diffuse Large B Cell Lymphoma (DLBCL) | [1]257 | [1]18351 |
| Head and Neck Cancer | [50,53,54,55]134337 | [50,53,54,55]75275 |
| Hepatocellular Carcinoma | [15,104,106]11287 | [15,104,106]2032 |
| High-Grade Glioma | [3,8,49,51,149]87540 | [3,8,49,51,149]16334 |
| Leukemia (Acute Lymphoblastic) | [6]95764 | [6]5930 |
| Leukemia (Acute Myelogenous) | [7]61048 | [7]21450 |
| Lymphoma | [16,34]905678 | [16,34]82310 |
| Melanoma (Locally Advanced Cutaneous) | [17]107605 | [17]8683 |
| Melanoma (Metastatic) | [17]47824 | [17]3859 |
| Multiple Myeloma (Newly Diagnosed) | 0 | [2]32270 |
| Nasopharyngeal Carcinoma | [56]5390 | [56]327 |
| NSC Lung Cancer | [35,57]454469 | [57] 191646 |
| NSC Lung Cancer Stage 3 | [35,57,143]151490 | [57,143]63882 |
| Oral Cancer (Advanced) | ^[4]^250000 | [4]53000 |
| Ovarian Cancer (Platinum-Resistant) | [36,37,38]141150 | [36,37,38]13956 |
| Ovarian Cancer, Primary Peritoneal Cavity Cancer | [40]2290 | [40]240 |
| Pancreatic Cancer (Locally Advanced) | [39,150]22066 | [39,150]17031 |
| Prostate Cancer | [18]3110403 | [18]174650 |
| Prostate Cancer (Localized) | [18]2395010 | [18]134481 |
| Prostate Cancer (Metastatic Hormone-Refractory) | [18]186624 | [18]10479 |
| Prostate Cancer (Newly Diagnosed) | 0 | [18]174650 |
| Recurrent Glioblastoma | [8,49,51,149]64127 | [8,49,51,149]12120 |
| Relapsed and Refractory Multiple Myeloma (RRMM) | [41,82]48840 | [41,82]16280 |
| Squamous Cell Cancer of Head and Neck or Esophagus | [50,53,54,55]120903 | [50,53,54,55]67747 |
| Synovial Sarcoma | [43,90]7282 | [90]655 |

# APPENDIX. ADDITIONAL DETAILS OF ANALYSIS

# A4 Calibration of Survival Functions *D_alt_*(*x*−*a*)

We source either the survival or mortality rate from literature and use them to compute *λ*, the time parameter in the exponential survival function. We show our result in the table below.

Table A4: List of survival rate or mortality rate and *λ*, for each disease. An asterisk (∗) under *λ* denotes that the disease does not affect mortality directly.

73

| Disease | *k* years survival rate | | *k* years mortality rate | | | *λ* |
| --- | --- | --- | --- | --- | --- | --- |
|  | *k* = 5 | *k* = 10 | *k* = 1 | *k* = 5 | *k* = 10 |  |
| Arteriosclerosis Obliterans |  |  |  | [162]11.3 |  | 0.024 |
| Critical Limb Ischemia |  |  |  | [87]50 |  | 0.139 |
| Degenerative Arthritis |  | [165]82 |  |  |  | 0.020 |
| Diabetic Foot Ulcers |  |  |  | [148]49 |  | 0.135 |
| Diabetic Peripheral Neuropathy |  |  |  |  | [62]5 | 0.005 |
| Heart Failure |  |  |  | [52]42.3 |  | 0.110 |
| Knee Osteoarthritis with Kellgren & Lawrence Grade 2 | [157]7.5 |  |  |  |  | 0.518 |
| Knee Osteoarthritis with Kellgren & Lawrence Grade 3 | [157]7.5 |  |  |  |  | 0.518 |
| Parkinson’s Disease | [167]40 |  |  |  |  | 0.174 |
| Peripheral Artery Disease |  |  |  | [76]33.2 |  | 0.081 |
| Refractory Angina due to Myocardial Ischemia (AFFIRM) |  |  | [111]3.9 |  |  | 0.040 |
| Stable Angina | [112]90 |  |  |  |  | 0.021 |
| Beta-Thalassemia |  | [171]98.3 |  |  |  | 0.002 |
| Cerebral Adrenoleukodystrophy (CALD) | [145]55 |  |  |  |  | 0.120 |
| Choroideremia Cystic Fibrosis |  |  |  |  | [122]28 | ∗  0.033 |
| Ewing’s Sarcoma | [133]70 |  |  |  |  | 0.071 |
| Hemophilia A |  | |  |  | [142]9.7 | 0.010 |
| Hemophilia B |  | |  |  | [142]9.7 | 0.010 |
| Leber Congenital Amaurosis due to RPE65 Mutations |  | |  | | |  |

∗

Continued on next page


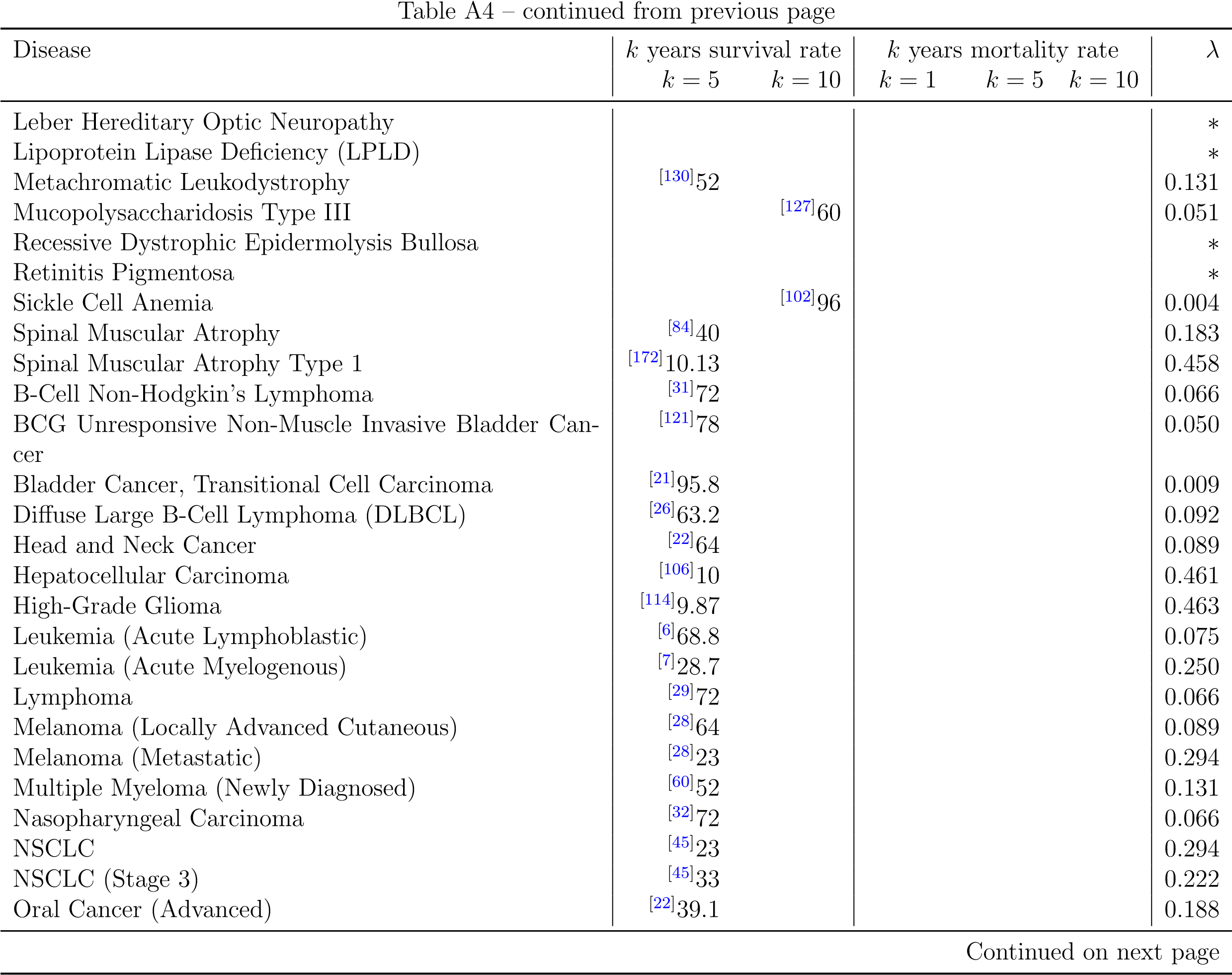


74

75

TableA4 –continued from previous page

Disease

*k*

Years survival rate

*k*

Years mortality rate

*λ*

*k*

=5

*k*

=10

*k*

=1

*k*

=5

*k*

=10

Ovarian Cancer (Platinum-Resistant)

[

164

]

1.9

0.793

Ovarian Cancer, Primary Peritoneal Cavity Cancer

[

23

]

47.6

0.148

Pancreatic Cancer (Locally Advanced)

[

24

]

12.4

0.417

Prostate Cancer

[

25

]

98

0.004

Prostate Cancer (Localized)

[

25

]

98

0.004

Prostate Cancer (Metastatic Hormone Refractory)

[

25

]

30.5

0.237

Prostate Cancer (NewlyDiagnosed)

[

25

]

95.1

0.010

Recurrent Glioblastoma

[

124

]

10

0.461

Relapsed and Refractory Multiple Myeloma (RRMM)

[

46

]

9.92

0.462

Squamous Cell Cancer of Head and Neck or Esophagus

[

22

]

64

0.089

Synovial Sarcoma

[

33

]

55

0.120

# A5 Calibration of Age Distribution *A*(*x*)

Our optimization program produces triangular age distributions that conforms to data, have wider support compared to fitting uniform distributions, and avoids sharp changes in the probability density. We illustrate some examples that compare triangle distributions with the uniform distributions with the same average age.

·10−^2^ ·10−^2^

0

2

4

6

8

10

0

1

2

3

0 20 40 60 80 100 0 20 40 60 80 100

(a) Comparative probability distributions when *µ_age_* = 10

(b) Comparative probability distributions when *µ_age_* = 30

·10−^2^ ·10−^2^

0

0

*.*

5

1

1

*.*

5

2

0

1

2

3

0 20 40 60 80 100 0 20 40 60 80 100

(c) Comparative probability distributions when *µ_age_* = 50 tions

(d) Comparative probability distributions when *µ_age_* = 80

Figure A5: Age distributions given various mean ages, *µ_age_*. The red triangles represent the solutions obtained by our optimization program, while the blue rectangles represent the solutions given by an uniform distribution. The distributions from the optimization program have a wider base of support and avoid sharp changes in density.

**A6 Quality of Life Estimation.**

Table of disease scores (*ζ*), estimated quality of life values before treatment *f*ˆ*_h_*(*s_alt_*), after treatment *f*ˆ*_h_*(*s_gt_*), and the change in quality of life (∆QoL). Asterisks (∗) indicate that the values are interpolated. Cancers are not included, as we assume that the gains in survival dominate the gains in QoL.

The results of our literature search and estimation for the change in QoL for each disease is shown in the table below.


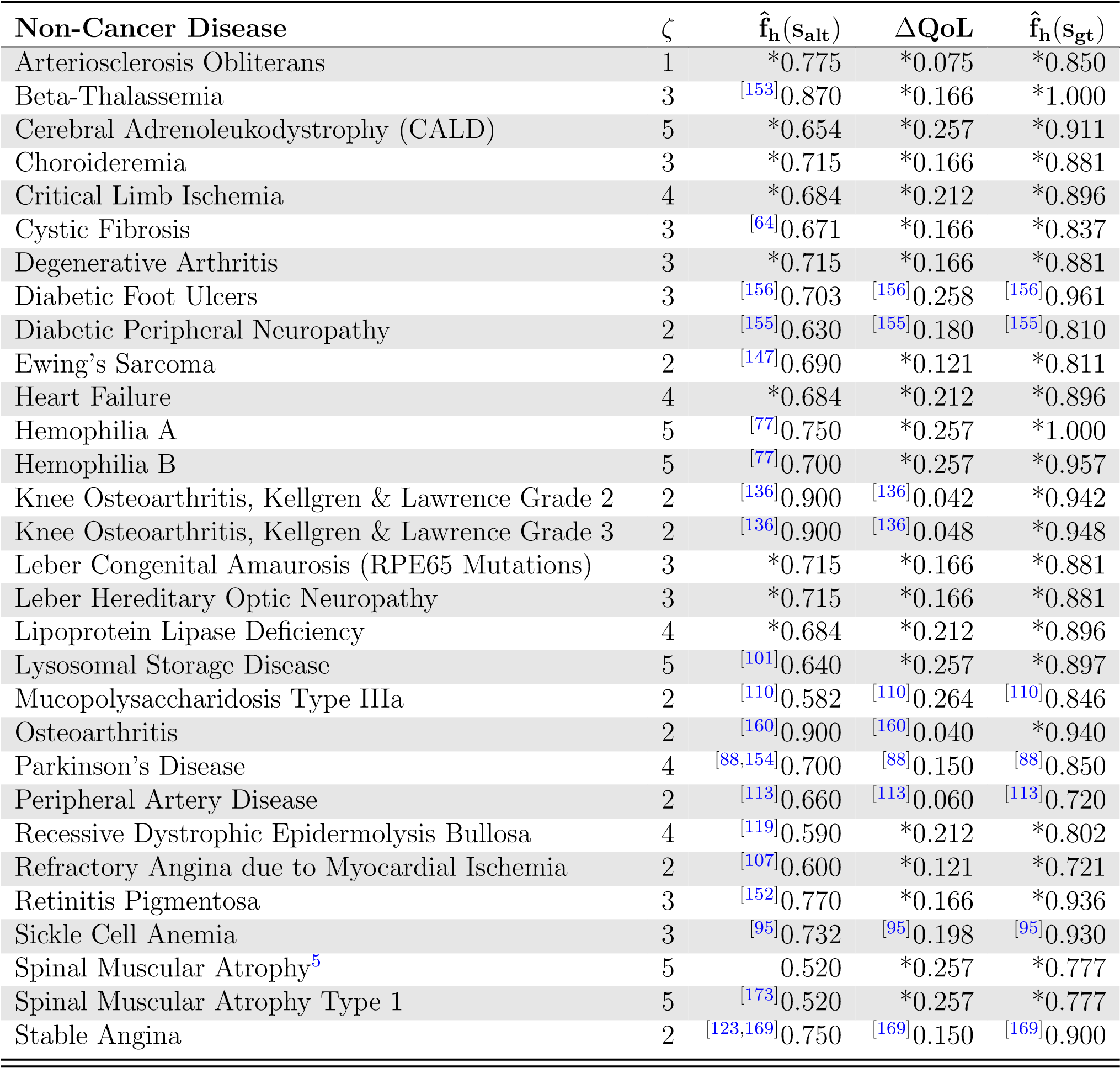


^5^We are unable to find QoL values for SMA only and assume that they are the same as SMA Type 1.

**A7 Estimated ∆QALY, assumed price per ∆QALY and estimated price of gene therapies per disease.**

Prices are given to 3 significant figures for display in this table.

## Disease ∆QALY ∆_QALY_^Cost^ ($) Price ($)

| **General Diseases:** |  |  |  |
| --- | --- | --- | --- |
| Arteriosclerosis Obliterans | 2.96 | 41K | 121K |
| Critical Limb Ischemia | 7.32 | 41K | 299K |
| Degenerative Arthritis | 3.53 | 41K | 144K |
| Diabetic Foot Ulcers | 7.92 | 41K | 323K |
| Diabetic Peripheral Neuropathy | 3.95 | 41K | 161K |
| Heart Failure | 6.92 | 41K | 282K |
| Knee Osteoarthritis with Kellgren & Lawrence Grade 3 | 10.62 | 41K | 433K |
| Parkinson’s Disease | 8.26 | 41K | 337K |
| Peripheral Artery Disease | 4.52 | 41K | 184K |
| Refractory Angina due to Myocardial Ischemia (AFFIRM) | 3.80 | 41K | 155K |
| Stable Angina | 3.85 | 41K | 157K |
| **Rare Diseases:** |  |  |  |
| Beta-Thalassemia | 4.58 | 102K | 466K |
| Cerebral Adrenoleukodystrophy (CALD) | 20.33 | 102K | 2.07M |
| Choroideremia | 4.24 | 102K | 431K |
| Cystic Fibrosis | 13.20 | 102K | 1.34M |
| Ewing’s Sarcoma | 14.04 | 102K | 1.43M |
| Hemophilia A | 11.18 | 102K | 1.14M |
| Hemophilia B | 10.63 | 102K | 1.08M |
| Leber Congenital Amaurosis due to RPE65 Mutations | 4.63 | 102K | 470K |
| Leber Hereditary Optic Neuropathy | 3.97 | 102K | 404K |
| Lipoprotein Lipase Deficiency (LPLD) | 5.74 | 102K | 584K |
| Metachromatic Leukodystrophy | 21.06 | 102K | 2.14M |
| Mucopolysaccharidosis Type IIIa | 16.27 | 102K | 1.65M |
| Recessive Dystrophic Epidermolysis Bullosa | 5.89 | 102K | 599K |
| Retinitis Pigmentosa | 3.28 | 102K | 333K |
| Sickle Cell Anemia | 7.36 | 102K | 748K |
| Spinal Muscular Atrophy | 19.23 | 102K | 1.96M |
| Spinal Muscular Atrophy Type 1 | 20.56 | 102K | 2.09M |
| **Cancer:** |  |  |  |
| B-Cell Non-Hodgkin’s Lymphoma | 4.90 | 41K | 200K |
| BCG Unresponsive NMIBC | 2.86 | 41K | 117K |
| Bladder Cancer, in situ concurrent with Papillary Tumors | 0.66 | 41K | 26.9K |
|  |  |  | |

**Disease**

**∆**

**QALY**

**Cost**

**∆**

**QALY**

**(**

$

**)**

**Price(**

$

**)**

| Diffuse Large B Cell Lymphoma (DLBCL) | 6.19 | 41K | 253K |
| --- | --- | --- | --- |
| Head and Neck Cancer | 6.13 | 41K | 250K |
| Hepatocellular Carcinoma | 9.30 | 41K | 380K |
| High-Grade Glioma | 12.56 | 41K | 512K |
| Leukemia (Acute Lymphoblastic) | 13.04 | 41K | 532K |
| Leukemia (Acute Myelogenous) | 8.55 | 41K | 349K |
| Lymphoma | 4.90 | 41K | 200K |
| Melanoma (Locally Advanced Cutaneous) | 6.23 | 41K | 254K |
| Melanoma (Metastatic) | 9.22 | 41K | 376K |
| Multiple Myeloma (Newly Diagnosed) | 5.90 | 41K | 241K |
| Nasopharyngeal Carcinoma | 5.20 | 41K | 212K |
| NSC Lung Cancer | 7.04 | 41K | 287K |
| NSC Lung Cancer Stage 3 | 6.52 | 41K | 266K |
| Oral Cancer (Advanced) | 8.21 | 41K | 335K |
| Ovarian Cancer (Platinum-Resistant) | 10.83 | 41K | 442K |
| Ovarian Cancer, Primary Peritoneal Cavity Cancer | 7.93 | 41K | 324K |
| Pancreatic Cancer (Locally Advanced) | 7.64 | 41K | 312K |
| Prostate Cancer | 0.42 | 41K | 17.1K |
| Prostate Cancer (Localized) | 0.42 | 41K | 17.1K |
| Prostate Cancer (Metastatic Hormone-Refractory) | 7.75 | 41K | 316K |
| Prostate Cancer (Newly Diagnosed) | 0.99 | 41K | 40.4K |
| Recurrent Glioblastoma | 12.55 | 41K | 512K |
| Relapsed and Refractory Multiple Myeloma (RRMM) | 8.33 | 41K | 340K |
| Squamous Cell Cancer of Head and Neck or Esophagus | 5.51 | 41K | 225K |
| Synovial Sarcoma | 8.65 | 41K | 353K |
|  | | | |

# A8 Simulation Convergence Criteria

Let *X_k_* be the results of the *k*-th simulation. *X_k_* has a true mean of *µ* and variance *σ*^2^. Let the mean of the Monte Carlo simulations over *n* runs be ˆ
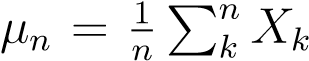
 . Then, by Lindeberg–L´evy’s Central Limit Theorem, ˆ*µ_n_* converges in distribution to a normal distribution with mean *µ* and variance of *nσ*^2^. The 95 percent confidence interval for *µ* is given by:


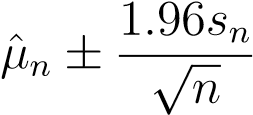
 (27)

where *s_n_* is the sample variance of {*X*_1_*,*··· *,X_n_*}.

Since we are using 1-by-T vectors, we investigated the error in our simulation by dividing the half-range of the confidence interval in each time-step by ˆ*µ_n_* before taking the maximum across the time series. As can be seen from Figure A2, we should expect the simulated mean to be within 1.89% of the true mean 95% of the time with 1,000,000 iterations.

10

4

10

5

10

6

0

*.*

000

0

*.*

002

0

*.*

004

0

*.*

006

0

*.*

008

0

*.*

010

1

*.*

96

*s*

*n*

*µ*

√

*n*

Number of runs

Plot of
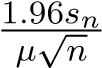
 against the number of iterations of simulations of the cost.

# A9 Pseudo-Code and Implementation Details

## Pseudo-code

We perform a Monte Carlo simulation to determine the total number of patients undergoing gene therapy and the cost of these gene therapies at specific points in time. The sequence of computations for each iteration of the simulation is detailed in Algorithm 1.

**Input :** D: A list of diseases

**Output:** Arrays of [1 × *T*], where *T* is the number of time steps.

P: Number of patients over time

C: Total cost over time

P ← 1 × T array of zeros C ← 1 × T array of zeros **for** *d* in D **do** p ← getPoS(d) // Get probability of success **if** random.uniform(0,1) ≤ *p* **then**

// If the disease gets an approval...

existing ← getExistingPatients(d) ; // Get existing patients (1 ×

T) new ← getNewPatients(d)// Get new patients (1 × T) *ρ* ← getRampFunction(d)// Get penetration ramp (1 × T) price ← getPrice(d)// Get price of GT (scalar) P+ = (existing + new)⊗*ρ* // Store number of patients

C+ = P× price// Store cost of treatment **end**

**end return** P*,* C

**Algorithm 1:** Pseudocode for one iteration of the simulation.

## Implementation

All the equations are discretized for computation from their continuous forms. When solving the integrals using the trapezoidal rule to obtain ∆QALY, we use strip widths of 1 year across a range from 0 to 110 years old, the resolution offered by the life tables. When simulating the number of patients and the cost over time, we use steps of 1 month.

Our codes are implemented on Python 3.6 backed by Numpy. Our vectorized implementation averages 6.120ms per iteration over 1,000,000 runs on a single thread of an Intel Xeon Gold 5120, clocked at 2.20GHz with 20GB of RAM. We attempted to use PyTorch to speed up the computations using a GPU, but it ran more slowly than a single-threaded CPU. We determined this took place for two reasons. First, generating random numbers must be sequential, since PyTorch delegates it solely to the CPU, which limits the amount of parallelization that can be achieved, as dictated by Amdahl’s law. Second, because our computations require a large amount of data from different sources, they must be batched due to the GPU’s limited RAM. The constant movement of data through the PCIe bridge, however, turns out to be a massive bottleneck to the overall speed.

# A10 Visualization of the Cost over Time

In this section, we visualize how the monthly cost of treating patients with gene therapy will be affected by changes to the variables. The results are summarized in the tornado chart presented in the main paper.

Jan2020

Jan2021

Jan2022

Jan2023

Jan2024

Jan2025

Jan2026

Jan2027

Jan2028

Jan2029

Jan2030

Jan2031

Jan2032

Jan2033

Jan2034

Jan2035

0

1

2

3

4

5

·

10

9

Cost

-20

%Phase3toBLAtime

+20

%Phase3toBLAtime

Baseline

Figure: Impact on monthly cost of treating patients given a ±20% change in the time from phase 3 to BLA.

Jan2020

Jan2021

Jan2022

Jan2023

Jan2024

Jan2025

Jan2026

Jan2027

Jan2028

Jan2029

Jan2030

Jan2031

Jan2032

Jan2033

Jan2034

Jan2035

0

1

2

3

4

5

·

10

9

Cost

-20

%BLAtoapprovaltime

+20

%BLAtoapprovaltime

Baseline

Figure: Impact on monthly cost of treating patients given a ±20% change in the time from BLA to approval.

Jan2020

Jan2021

Jan2022

Jan2023

Jan2024

Jan2025

Jan2026

Jan2027

Jan2028

Jan2029

Jan2030

Jan2031

Jan2032

Jan2033

Jan2034

Jan2035

0

1

2

3

4

5

·

10

9

Cost

-20

%Existingpatients

+20

%Existingpatients

Baseline

Figure: Impact on monthly cost of treating patients given a

±

20

% change in the number of existing patients

of existing patients.

Jan2020

Jan2021

Jan2022

Jan2023

Jan2024

Jan2025

Jan2026

Jan2027

Jan2028

Jan2029

Jan2030

Jan2031

Jan2032

Jan2033

Jan2034

Jan2035

0

1

2

3

4

5

·

10

9

Cost

-20

%Newpatients

+20

%Newpatients

Baseline

Figure: Impact on monthly cost of treating patients given a

±

20

%change in the number of new patients

Jan2020

Jan2021

Jan2022

Jan2023

Jan2024

Jan2025

Jan2026

Jan2027

Jan2028

Jan2029

Jan2030

Jan2031

Jan2032

Jan2033

Jan2034

Jan2035

0

1

2

3

4

5

·

10

9

Cost

-20

%PoS

3

*A*

+20

%PoS

3

*A*

Baseline

Figure: Impact on monthly cost of treating patients given a

±

20

% change in the PoS.

3

*A*

.

Jan2020

Jan2021

Jan2022

Jan2023

Jan2024

Jan2025

Jan2026

Jan2027

Jan2028

Jan2029

Jan2030

Jan2031

Jan2032

Jan2033

Jan2034

Jan2035

0

1

2

3

4

5

·

10

9

Cost

%∆QALY

-20

+20

%∆QALY

Baseline

Figure: Impact on monthly cost of treating patients given a

±

20

% change in ∆QALY

gained.

Jan2020

Jan2021

Jan2022

Jan2023

Jan2024

Jan2025

Jan2026

Jan2027

Jan2028

Jan2029

Jan2030

Jan2031

Jan2032

Jan2033

Jan2034

Jan2035

0

1

2

3

4

5

·

10

9

Cost

%Costper∆QALY

-20

+20

%Costper∆QALY

Baseline

Figure: Impact on monthly cost of treating patients given a

±

20

%change in the cost

Per ∆QALY.

Jan2020

Jan2021

Jan2022

Jan2023

Jan2024

Jan2025

Jan2026

Jan2027

Jan2028

Jan2029

Jan2030

Jan2031

Jan2032

Jan2033

Jan2034

Jan2035

0

1

2

3

4

5

·

10

9

Cost

-20

%Θ

*max*

+20

%Θ

*max*

Baseline

Figure: Impact on monthly cost of treating patients given a ±20% change in Θ*_max_*.

Jan2020

Jan2021

Jan2022

Jan2023

Jan2024

Jan2025

Jan2026

Jan2027

Jan2028

Jan2029

Jan2030

Jan2031

Jan2032

Jan2033

Jan2034

Jan2035

0

1

2

3

4

5

·

10

9

Cost

-20

%

*T*

*max*

+20

%

*T*

*max*

Baseline

Figure A11: Impact on monthly cost of treating patients given a ±20% change in *T_max_*.
